# Chemically Selective Nanoelectrode Arrays for Real‐Time, Parallel Neurotransmitter and Electrical Recording

**DOI:** 10.1002/smsc.70249

**Published:** 2026-03-28

**Authors:** Shivani Shukla, An‐Yi Chang, Anum Tahir, Muhammad Inam Khan, Maria Reynoso, Ashley Pham, Yuma Dugas, Ian McGregor, Nawab John Dar, Noel Sebastien D Mallari, Dhivya Pushpa Meganathan, Adam T. Woolley, Joseph Wang, Zeinab Jahed

**Affiliations:** ^1^ Aiiso Yufeng Li Family Department of Chemical and Nano Engineering University of California San Diego La Jolla California USA; ^2^ Shu Chien‐Gene Lay Department of Bioengineering University of California San Diego La Jolla California USA; ^3^ Chan Zuckerberg Biohub Chicago Chicago Illinois USA; ^4^ Department of Chemistry and Biochemistry Brigham Young University Provo Utah USA; ^5^ Department of Cellular Neurobiology The Salk Institute for Biological Studies La Jolla California USA

## Abstract

Electrical activity and neurotransmitter release are tightly coupled in neurons, but co‐registered measurements of both modalities from the same subcellular site in real time have remained difficult. Bridging this gap is essential for linking single‐cell processing to network dynamics and for enabling closed‐loop neural interfaces. Here, we introduce Graph‐nanoelectrode arrays (NEAs), graphite‐modified nano‐electrode arrays that unify intracellular‐like electrophysiology with electrochemical neurotransmitter sensing. Graph‐NEAs record supra‐ and sub‐threshold electrical activity together with dopamine release currents from sub‐neuronal locations in live neuron‐like networks, with high sensitivity, selectivity, and stability. Using a single multimodal setup, we validate chemical readouts against calcium imaging and electrical signals and show that dopamine dynamics closely track sub‐threshold electrical activity across seconds with electrical stimulation or potassium chloride, and across minutes with vesicular transporter inhibition by reserpine. We further recapitulate Parkinson's‐relevant oxidative stress through chronic glutathione depletion using buthionine sulfoximine or prolonged iron exposure. An optical synaptic vesicle assay confirms that the chemical currents originate from neurotransmitter release at the cell–nanoelectrode interface. By unifying chemical and electrical sensing within the same nanoscale platform, Graph‐NEAs establish a new paradigm for multimodal neural recording with applications in closed‐loop neuromodulation, disease modelling, drug discovery, and neuromorphic engineering.

## Graphical abstract

In this work, the authors demonstrate proof‐of‐concept, multimodal recordings of dopamine and intracellular electrical activity from differentiated SH‐SY5Y neuronal networks using graphite‐modified nanopillar electrode arrays. This study paves the way for multimodal, single cell experiments on neuronal networks to elucidate the dynamics between electrical activity and chemical neurotransmission.

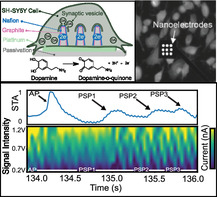

## 1 Introduction

Electrical activity and neurotransmitter release are tightly coupled in neurons, yet co‐registered measurements of both from the same subcellular site have been difficult. Resolving this gap is important for linking single‐cell processes to network dynamics and for building closed‐loop neural interfaces [1, 2, 3, 4, 5]. Existing tools [6, 7, 8, 9, 10, 11, 12, 13] while offering rapid electrochemical [14, 15, 16] or electrophysiological detection [17, 18, 19] typically capture one modality at a time or lack the spatial precision to relate single‐to‐few neurotransmitter release events to local membrane potentials. Toward this goal, subcellular electrodes offer a promising solution to create closed‐loop feedback control [20, 21] at the synaptic level [2].

Nanoelectrode arrays (NEAs) have established intracellular‐like electrical access at subcellular resolution and have been used to map functional connectivity and estimate synaptic outputs in cultured neuronal networks (Figure 1a,b) [24, 25, 26]. Across multiple implementations, NEAs resolve sub‐threshold postsynaptic events and action potentials, enabling synaptic mapping in 2D and 3D models [24, 25, 27, 28, 29, 30, 31, 32, 33, 34, 35, 36].

FIGURE 1Subcellular electrical and electrochemical sensing using graphite‐modified nanopillar electrode arrays (Graph‐NEA). (a) Schematic of the Graph‐NEA device containing 60 individually addressable nanoelectrodes. (b) Brightfield image of the device showing the 60‐electrode array, possessing 58 35 × 35 µm electrodes, and 2 longer arc electrodes for eventual multicellular stimulation. (c) Schematic of a single nanoelectrode containing 9 nanopillars. (d) Scanning electron micrographs of an individual nanopillar before (i) and after (ii) the graphite/Nafion coating (scale bar, 500 nm). (e) Schematic illustrating the layer‐by‐layer coating strategy that enables nanopillars to sense changes in dopamine concentration via electrochemical detection. (f) Cyclic voltammetric response to 5 µM dopamine on bare platinum, graphite, and Nafion‐coated nanopillars. (g) *In vitro* cyclic voltammetric dopamine detection on Graph‐NEA in PBS across concentrations from 0–25 µM. Inset shows linear calibration for estimating concentrations measured during live cell recording. (h) Five representative sensing channels demonstrating reproducibility across devices. (i) Bar plot quantifying selectivity of dopamine detection compared to other biomolecules, based on cyclic voltammetry peak currents. Error bar represents standard deviation (SD). (j) Top: schematic of nanoelectrode interfaced with SH‐SY5Y neuroblastoma cells. Bottom: colorized scanning electron micrograph showing an SH‐SY5Y cell (pseudo‐colored green) engulfing nine Graphite‐coated nanopillars within a single nanoelectrode. (k) Schematic of the sequence of electrical and electrochemical events at the cell–nanoelectrode interface. (i) The neuron is at rest, with dopamine and similar neurotransmitters loaded into the synaptic vesicle reserve pool. (ii) The cell receives a signal from an external stimulus, or a synaptic input from a nearby cell via ligand‐gated sodium channels, causing a sub‐threshold potential. (iii) The signal causing the sub‐threshold potential is strong and/or frequent enough to pass the depolarization threshold, causing voltage‐gated sodium channels to open across the cell as the supra‐threshold action potential travels to the presynaptic terminal. (iv) At the presynaptic terminal, the cell's depolarized membrane causes the voltage‐gated calcium channels to open. (v) At the presynaptic terminal, the influx of calcium triggers a biological cascade of synaptic proteins to allow synaptic vesicle fusion to the plasma membrane followed by neurotransmitter release into the synaptic cleft [22]. This neurotransmitter release is measured by well‐coupled nanoelectrodes as the dopamine release current. (l) Schematic of a cell on a Graph‐NEA depicting dopamine electrochemical reactions. (m) Top: Spike‐triggered average (STA) of electrical responses from neighboring channels in an SH‐SY5Y neuronal network. Bottom: heatmap of current responses from cyclic voltammetry over time. (n) Bar plot showing cell viability on Pt‐NEAs (nonmodified), and Graph‐NEAs with error bar showing relative standard deviation (RSD). All characterization and viability experiments were performed with 3–4 Graph‐modified nanoelectrode arrays and unmodified nanoelectrode arrays for viability experiments, at least 3 times. Graph‐NEA maintained cell viability above 70%, corroborated by previous SH‐SY5Y dopamine sensors [23], and showed no significant difference compared to unmodified NEA (*p* = 0.124).

**Figure:**
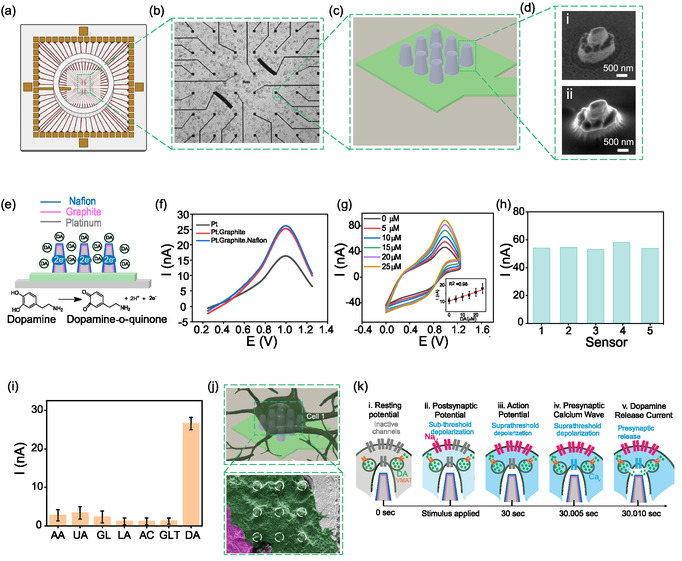

**Figure:**
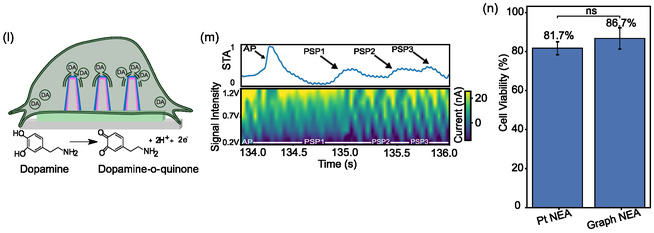

Despite these advances, NEA recordings remain purely electrical and lack a co‐registered, chemically selective readout of neurotransmitters, leaving synaptic release inferred rather than directly measured.

Measuring neurotransmitter concentrations near the synaptic cleft would directly relate electrical events to quantal release, with single‐vesicle content serving as a metric of synaptic strength [1, 11]. Carbon‐based and conductive‐polymer electrodes provide the sensitivity, selectivity, and antifouling properties needed for electrochemical detection of micromolar neurotransmitter concentrations over extended periods [22, 37, 38, 39, 40, 41, 42], but stably miniaturizing such chemistries to interface with single cells at synaptic distances and integrating them with subcellular electrical access have not been achieved.

Here, we introduce Graph‐NEAs, graphite‐modified nanopillar electrode arrays that combine intracellular‐like electrophysiology with electrochemical neurotransmitter sensing at the same subcellular site. Graph‐NEAs simultaneously record supra‐ and sub‐threshold electrical activity together with dopamine release current signals in live neuron‐like networks (Figure 1). The chemical dopamine readouts are validated against calcium imaging and electrical signals within a single multimodal setup. We further demonstrate that both sensing modalities respond in concert to controlled perturbations across seconds to minutes, including electrical stimulation and potassium chloride depolarization, vesicular transporter inhibition by reserpine and redox challenges produced by chronic buthionine sulfoximine (BSO) treatment, and chronic iron modulation. In these redox‐shifts conditions, Graph‐NEAs resolve predictable changes in dopamine oxidation current amplitude and kinetics, consistent with altered vesicle filling/release probability and enhanced catechol oxidation. An optical synaptic vesicle assay confirms that the chemical currents originate from vesicle release at the cell–nanoelectrode interface. We show that the Graph‐NEAs can detect dopamine within the well‐documented range of 0–30 µM expected of differentiated SH‐SY5Y cells [23, 37, 38, 39, 43]. We estimate a limit of detection around ~3.87 µM, which echoes the results of previous dopamine nano‐biosensors with ~500 nm sensing diameter [40, 41, 43], and suggests that Graph‐NEA sensors record multiple dopamine release events at once from coupled overlying cells within the differentiated SH‐SY5Y network [42]. Graph‐NEAs therefore offer a multiplexed platform for recording multimodal electrical and electrochemical signals from electrogenic cellular networks.

## 2 Results and Discussion

### 2.1 Design, Fabrication and Characterization of Graph‐NEAs

We developed Graph‐NEAs, graphite‐modified platinum NEA that co‐locate intracellular‐like electrical access with chemically selective dopamine sensing on the same device (Figure 1). Each device integrates up to 60 individually addressable nanoelectrodes (Figure 1a,b and Supplementary Figure 1); each nanoelectrode comprises a 3 × 3 square array of platinum nanopillars with tip radius ~250 nm, base radius ~500 nm, and height ~1 µm before coating (Figure 1c–d and Supplementary Figure 1). Following the fabrication protocol previously reported by our group [24], graphite and Nafion were conformally applied by sequential electrodeposition, which slightly increased the effective tip radius while preserving pillar geometry (Figure 1d(i–ii),e and Supplementary Figure 2). Both the electrochemical impedance spectrum and impulse response of Graph‐NEAs matched closely with bare NEAs previously fabricated by our lab (Supplementary Figure 3) [24]. The platform supports simultaneous optical imaging (bright‐field and fluorescence calcium imaging), electrophysiology, and electrochemistry in the same field of view without loss of signal integrity, as shown schematically in Supplementary Figure 4.

Our ***Graph‐NEAs*** offer selectivity and sensitivity to dopamine. Graphite provides catecholamine sensitivity, and Nafion improves stability and antifouling, yielding clear oxidation peaks in cyclic voltammetry (CV) (Figure 1f,g), aligning with reported values in literature where graphite and other carbon‐based electrodes have been combined with Nafion for dopamine detection [44, 45, 46, 47]. Graph‐NEAs detect dopamine over 0–25 µM in phosphate‐buffered saline (PBS) using the oxidation peak for quantification, with peak potentials between 0.8 and 1.0 V depending on electrode properties (Figure 1g) [14]. Responses are stable over repeated cycles up to 30 s and reproducible across sensors (Figure 1h and Supplementary Figure 5). Selectivity assays show roughly tenfold higher current for dopamine than for uric acid, glutamate, lactic acid, glucose, acetaminophen or ascorbic acid (Figure 1i), and a linear calibration enables estimation of relative concentration changes during live‐cell recording (Figure 1g, inset). The ~20 ms CV sampling interval aligns with dopamine release dynamics.

We next evaluated the sensitivity of the Graph‐NEAs in SH‐SY5Y culture media containing serum and observed that the high current response to dopamine was preserved (Supplementary Figure 6a). These results indicate that the presence of serum proteins and complex media components does not substantially attenuate dopamine detection. Having first validated the Graph‐NEAs under cell‐free conditions, we next performed real‐time dopamine measurements in cellular models widely used to study dopaminergic and electrophysiological dynamics. Differentiation of SH‐SY5Y cells using retinoic acid has been shown to produce predominantly dopaminergic networks [48], making this cell type a biologically relevant model for evaluating dopamine sensing using the Graph‐NEAs. Accordingly, we used differentiated SH‐SY5Y cells to probe real‐time catecholamine release in a system that captures both dopaminergic signaling and electrical network dynamics. In vitro characterization revealed that the current response to dopamine was not significantly greater than that of other catecholamines under the tested conditions (Supplementary Figure 6b), indicating limited chemical selectivity of the Graph‐NEAs in their current form. Because we lacked a direct, multimodal approach to unambiguously identify the specific catecholamine released during cellular measurements, we view improved chemical discrimination as an important direction for future development.

When a neuron tightly engulfs the nanoelectrodes (Figure 1j), Graph‐NEAs provide sub‐ and supra‐threshold electrical readouts while simultaneously sensing local dopamine release. Figure 1k summarizes the expected sequence on a common timeline: following resting potential (Figure 1ki), an external stimulus is registered on the tightly coupled nanoelectrode as a subthreshold potential (Figure 1kii). This is followed within tens of milliseconds by suprathreshold action potentials (Figure 1kiii); subsequent Ca^2+^ entry generates a calcium wave (Figure 1kiv), and vesicular dopamine released adjacent to the nanoelectrode is oxidized on the graphite/Nafion nanoelectrode surface (Figure 1kv), producing a current response that reflects relative changes in dopamine (Figure 1l‐m). These events occur within our ~20 ms CV sampling window and are co‐registered with electrical and optical readouts in the same field of view.

### 2.2 Graph‐NEAs Capture Electrically Induced Changes in Dopamine Response and Electrical Activity

With the acellular performance established, we performed real‐time multimodal recordings in SH‐SY5Y neuron‐like cells. This catecholaminergic line shows reliable dopamine release in disease models [49, 50, 51], robust electrical signaling including postsynaptic potentials (PSPs) and action potentials (APs) by patch clamp [52, 53, 54, 55, 56], and maintains a soma large enough to engulf a 3 × 3 pillar cluster after differentiation [48, 56, 57, 58]. The cell viability on Graph‐NEAs was not significantly different than that on the glass and Pt controls (Figure 1n and Supplementary Figure 7).

**FIGURE 2:**
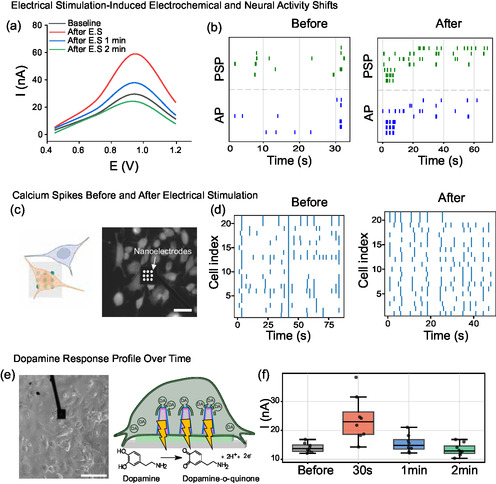
Electrical stimulation enables multi‐scale electrical and electrochemical recording. (a) Cyclic voltammogram showing current peak in response to electrical pulse over time on graph‐modified NEAs with SH‐SY5Y cells. (b) Representative raster plot of supra ‐and sub‐threshold events before and after electrical stimulation. (c) Left: Schematic showing single neuron recording from single nanoelectrode. Right: Representative maximum intensity projection showing Fluo‐4 AM inside SH‐SY5Y cells surrounding Graph‐modified nanoelectrode (scale bar = 35 µm). (d) Raster plot for 20 SH‐SY5Y cells of calcium spikes before and after electrical stimulation, respectively. Before stimulation, there are only 2 “synchronous events” where most of the cells fire around the same time. After stimulation, there are roughly 6–7 synchronous events where > 10 cells fire around the same time. (e) Left: Brightfield image of SH‐SY5Y cells . Right: Schematic demonstrating mechanism of increased dopamine release following an electrical pulse through the nanopillars (scale bar = 1 µm). (f) Bar plot showing the dopamine response profile over time following the electrical stimulation. All electrical stimulation experiments were performed with 3–4 graphite and Nafion‐modified nanoelectrode arrays during 6 independent experiments.

As an initial functional validation of the Graph‐NEAs, we used electrical stimulation as a positive control known to increase neuronal excitability and evoke dopamine release. We delivered a brief pulse through the targeted nanoelectrode and recorded dopamine and electrical activity from neighboring channels while imaging calcium to confirm activation. Within ~30 s of stimulation, the dopamine oxidation current increased (Figure 2a) and coincided with a transient rise in electrical activity containing a mix of both sub‐threshold, postsynaptic potentials and action potentials (Supplementary Figure 8). We quantified how stimulation propagates across the network from sub‐neuronal interface to single cells and the surrounding network. Raster plots and interspike interval distributions are summarized in Figure 2b and Supplementary Figure 9, showing significantly lower (*p* < 0.05) intervals for both action potentials and postsynaptic potentials. After electrically stimulating a targeted cell, the underlying channel exhibits increased dopamine release, while raster plots reveal higher rates of PSPs and supra‐threshold APs across active channels. Electrical events were parsed by dynamic spike sorting as previously described [59]. As a control, HL‐1 cardiomyocyte‐like cells displayed electrical and calcium activity without a dopamine CV signature, and SH‐SY5Y cultures exhibited an approximately tenfold larger CV oxidation response than HL‐1 (Supplementary Figures 10 and 11) both with and without electrical stimulation.

Consistent with the network‐wide rise in PSPs and APs shown in Figure 2b, we next asked whether this electrical propagation recruits nearby cells at the population level. Figure 2c focuses on the stimulated region: the left schematic depicts a target cell on a nanoelectrode with neighboring cells; the right fluorescence image shows the cells neighboring the nanoelectrode during calcium imaging. Figure 2d shows raster plots of calcium spikes from 20 neighboring SH‐SY5Y cells before and after stimulation, revealing a clear increase in synchronous Ca^2+^ activity following the electrical stimulation. An independent samples t‐test revealed a highly significant difference between baseline and stimulation inter‐spike intervals for the calcium spikes (*p* << 0.01), indicating a robust stimulation‐induced change in neuronal firing dynamics (Supplementary Figure 12). Thus, both the focal electrical activity of single cells above single Graph‐NEA nanoelectrodes, as shown in the brightfield image in Figure 2e, and the neighboring SH‐SY5Y cellular network activity supports the observation of increased dopamine release in response to electrical stimulation.

Having established network‐wide electrical and calcium responses, we next quantified the dopamine time course across many cells. Figure 2e links the electrochemical readout to the cell–nanoelectrode release interface: schematics illustrate dopamine released adjacent to the engulfed tip diffusing through the narrow cell–electrode cleft, where it is oxidized on the Graph‐NEA surface. Figure 2f summarizes recordings across experiments, showing a transient increase in the oxidation current, peaking at ~30 s, partially recovering by 1 min, and trending toward baseline by 2 min. Together, these measurements establish electrical stimulation as a positive‐control validation of Graph‐NEA multimodal sensing and motivate an orthogonal, pharmacological test of chemical specificity and dynamic range.

### 2.3 Graph‐NEAs Capture Pharmacologically Induced Changes in Dopamine Response

To verify the biological origin of the dopamine signal and map the dynamic range across timescales, we perturbed SH‐SY5Y networks with agents that are known to modulate excitability and neurotransmitter release. Potassium chloride (KCl) lowers the depolarization threshold and transiently increases both postsynaptic and spiking activity [24, 60], which should elevate dopamine release. Reserpine inhibits VMAT2, blocking vesicular packaging of monoamines and thereby suppressing dopamine release with secondary effects on subthreshold activity [61, 62, 63]. Evidence of both KCl‐ and reserpine modulation of neuronal network activity has been well‐documented in literature. We also treated neuroblastoma cells with BSO and iron for a chronic oxidative stress model. BSO depletes glutathione and elevates oxidative stress. Glutathione reduction leads to dysfunction mitochondria and lower production and synthesis of new dopamine [64, 65]. Whereas chronic iron exposure reduces releasable vesicular dopamine, consistent with oxidative consumption and impaired vehicular loading [66, 67].

#### 2.3.1 KCl Depolarization (Seconds to *M*inutes)

**FIGURE 3:**
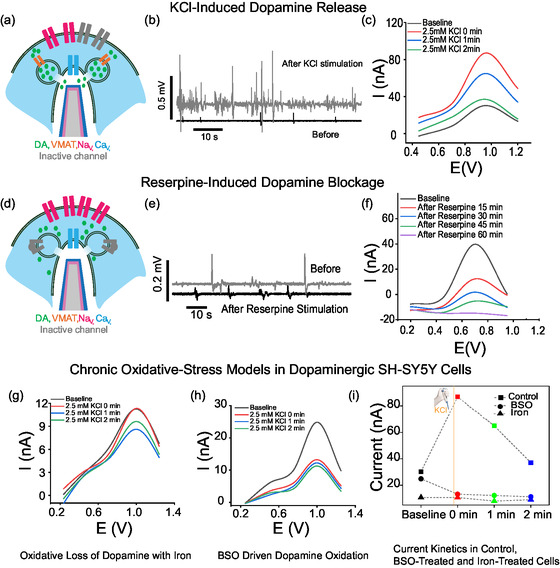
Chemical stimulation of dopamine release enables multiscale electrical and electrochemical recordings. (a) Schematic showing membrane close‐up during short‐term KCl‐induced depolarization. (b) Representative electrical recording trace before (black) and after (gray) KCl addition. (c) Cyclic voltammogram of SH‐SY5Y cell on single nanoelectrode in response to 2.5 mM KCl. (d) Schematic showing membrane close‐up during long‐term reserpine blockage of dopamine transport into synaptic vesicles. (e) Representative electrical recording trace before (gray) and after (black) reserpine addition. (f) Cyclic voltammograms over 1 h of SH‐SY5Y cell on single nanoelectrode measured inside the incubator in response to reserpine addition. (g) Cyclic voltammogram shows loss of dopamine in iron induced chronic oxidative loss of dopamine. (h) Cyclic voltammogram showing reduction of dopamine in chronic BSO oxidative model. (i) Current kinetics figures shows trend of baseline dopamine and after KCl stimulation in healthy neuroblastoma cells and chronic models; BSO and iron.

Stepwise KCl additions produced graded increases in electrical activity and in the dopamine oxidation peak (Figure 3a–c). Subthreshold postsynaptic potentials were more frequent and stratified after KCl addition compared with action potentials (Supplementary Figure 13a,b). The largest dopamine changes in Figure 3c coincided with the significantly shorter interspike intervals for both AP and PSP event classes around ~2.5 mM KCl, consistent with heightened excitability. The effect was transient and returned toward baseline within minutes (Supplementary Figure 14). With further KCl, both electrical and dopamine signals collapsed between 7 and 10 mM (see Supplementary Figure 15), consistent with loss of viability that depended on seeding density and coverage. This upper limit on KCl stimulation was unsurprising given the 50 mM concentration used with large substrates and perfusion experiments, representing diluted effects of KCl addition, reported in literature using the SH‐SY5Y cell line [68, 69, 70]. FM‐143 synaptic staining, presented in the next section, corroborated stimulation‐evoked vesicle release at the same sites.

#### 2.3.2 VMAT2 Inhibition by Reserpine (*M*inutes to Hours)

To suppress vesicular packaging and release, we applied reserpine (5 µM), a VMAT2 inhibitor that must be transported into the cell before acting (Figure 3d). Because the effect unfolds over minutes to hours, we used our multimodal setup inside the incubator at 37°C and 5% CO_2_ to preserve physiology during continuous recording of electrical activity and dopamine CV signals (optical readouts were not acquired concurrently). Relative to baseline, both electrical events and the dopamine oxidation current decreased within minutes (Figure 3d–f), with the largest drop in current occurring at ~ 15 min, as quantified by the statistically shorter interspike‐intervals for both supra‐threshold APs and sub‐threshold PSPs (Supplementary Figure 13c,d), and the minimum current detected around ~60 min. After ~60 min, signals partially recovered at this dose, indicating functional reversibility under our conditions. These data confirm that VMAT2 blockade reduces dopamine release with a matched decline in subthreshold excitability and that Graph‐NEAs resolve these pharmacologically driven changes on the expected timescale.

Taken together, KCl depolarization and VMAT2 blockade drive predictable, bidirectional changes in dopamine oxidation and matched shifts in subthreshold and spiking activity on seconds‐to‐minutes timescales. Furthermore, the increase (KCl) or decrease (reserpine) of positive spikes above a 1 mV threshold with 20 – 100 ms duration matches prior reports of intracellular‐like electrical recording using nanopillar electrodes [24, 25, 26, 71, 72] and verifies that the Graph‐NEA devices can reliably record electrical signals from SH‐SY5Y networks with spontaneous, intracellular‐like access, similar to unmodified NEA previously reported by our group [24].

#### 2.3.3 Chronic Oxidation by Iron and BSO

Iron and BSO treatment assays were used to further validate dopamine detection. SH‐SY5Y cells were exposed to iron or BSO every other day for 6 days. Chronic iron treatment impairs the cells’ ability to release dopamine. Baseline measurements show a reduction in dopamine signal compared with nontreated cells. After stimulation with KCl shown in Figure 3g, the chronically iron‐treated cells exhibit a response close (red trace, immediately upon KCl stimulation) to or below (green and blue traces, 1 and 2 min after stimulation, respectively) baseline that declines further over the next few minutes, in contrast with the increased response from KCl stimulation in non‐iron treated cells shown previously in Figure 3c. This severe impairment is consistent with iron's well‐documented ability to catalyze dopamine oxidation and generate harmful reactive oxygen species that damage dopaminergic neurons and their ability to regulate dopamine release [66, 67]. Contrastingly, BSO treatment impairs the maintenance of dopamine release machinery, causing a different signature in the dopamine current response, shown in Figure 3h. Upon stimulation (red trace, 0 min), the signal is reduced relative to baseline cells, suggesting that BSO‐induced glutathione depletion impairs the ability of dopaminergic cells to maintain and release dopamine, in line with studies showing that reduced brain glutathione potentiates dopamine‐depleting insults and dopaminergic dysfunction [64, 73]. After stimulation, because no new dopamine is being synthesized and released, the signal is lower than baseline and continues to decrease after 1 (blue trace) and 2 min (green trace). This pattern suggests dysregulation of cellular machinery, such as mitochondria. These findings align with previous studies showing that glutathione depletion increases the vulnerability of nigrostriatal/dopaminergic neurons to oxidative stress and perturbs normal dopamine homeostasis [64, 65, 73, 74]. Together, these results indicate that our Graph‐NEA sensor was able to sense signals from both oxidative stress models iron and BSO (Figure 3g–i)

### 2.4 Benchmarking Dopamine Sensing at Targeted Graph‐NEA Nanoelectrodes

**FIGURE 4:**
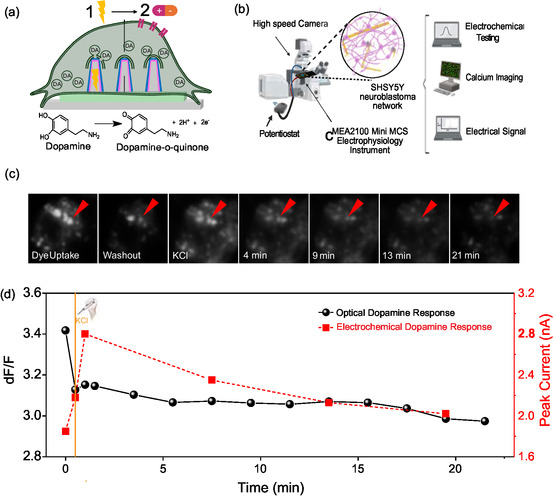
Sub‐neuronal biological mechanisms during Graph‐NEA recording. (a) Schematic illustrating how both electrical pulse and KCl were used to allow FM‐143 entry into synaptic vesicles and then stimulate their release. (b) Schematic showing a multimodal data acquisition setup for simultaneous electrical recording, dopamine sensing, and synaptic vesicle release imaging. (c) Time frame series showing synaptic vesicles release representative images of synaptic vesicle punctate releasing FM‐143 dye over time around the chosen nanoelectrode. (d) Representative signals overtime following KCl stimulation. Left axis: dF/F trace (black) from optical synaptic vesicle release assay showing stimulus evoked vesicle exocytosis overtime. Right axis: dopamine oxidation peak (red) from CV. All FM‐143 assays were performed with 2 graphite and Graph‐NEAs during 2 independent experiments.

To prove that synaptic vesicular release of dopamine is indeed the underlying mechanism behind our electrochemical measurements, we performed FM‐143 synaptic vesicle release assays on SH‐SY5Y neuronal networks during dopamine sensing and electrical recording. In accordance with previous literature [75, 76, 77], we achieved dye uptake and release by using a two‐step stimulation approach which combined both our electrical stimulation and KCl addition protocol (see schematic in Figure 4a), based on the protocol by Gaffield and Betz [78]. This assay demonstrates the active release of neurotransmitters from intracellular synaptic vesicles by first capturing the FM‐143 dye via induced endocytosis, which has been successfully demonstrated via electrical stimulation [79, 80, 81, 82, 83]. Next, activation of electrical activity is performed, such as by KCl stimulation, to induce release of both neurotransmitter and FM‐143 dye from the synaptic vesicles, and cause a decrease in fluorescence intensity within preidentified vesicle punctae over the course of several minutes. This order and method of double stimulation was chosen due to the body of research showing how high‐curvature nanostructures attract endocytic proteins [84, 85, 86], and thus using high‐curvature nanostructures to deliver electrical stimulation enabled enhanced endocytic events. After endocytosis of the FM1−43 dye, we chose to use KCl to effectively lower the depolarization threshold to trigger neurotransmitter release [43, 87]. Furthermore, we used this assay to demonstrate the utility of our multimodal setup (shown in Figure 4b) which can simultaneous record optical electrophysiological signals, dopamine response from up to 2 nanoelectrodes at the same time, and electrical recording of all neighboring nanoelectrodes on the same Graph‐NEA device.

The full FM‐143 assay showed clear neurotransmitter release from single SH‐SY5Y cells differentiated on Graph‐NEA devices. Figure 4c shows time frames of selected punctae close to the nanoelectrode having synaptic vesicle release over the course of the assay, with red triangles indicating the punctae which become less bright over time. The first three images in Figure 4c correspond to the first three black dots shown in the black trace of the line plot in Figure 4d. Specifically, when the dye is added via electrical stimulation, the punctae show high fluorescence intensity. This intensity drops when the dye is washed out, and then partially recovers once KCl is added to stimulate the cells to release dopamine (and also the dye) from internal stores. The black line plot in Figure 4d shows the FM‐143 release profile at punctae close to the nanoelectrode used for dopamine recording. At the same time, we recorded the dopamine response from this same nanoelectrode, which is summarized in the red line plot in Figure 4d. The initial drop in fluorescence intensity occurs after excess dye washout, followed by a small peak directly after a single KCl addition, and then a subsequent decrease in fluorescent intensity for the remaining 17 min. Additionally, Supplementary Figure 16 provides violin plots of punctae per cell cluster, and statistical analyses showing the signature dye uptake and stark drop in fluorescent signal after the second, KCl‐induced synaptic vesicle release over several minutes. We concluded that SH cells near and, on the Graph‐NEA electrodes were actively releasing neurotransmitter during the course of the assay. We saw this response in both the decreasing intensity of adjacent synaptic vesicles to the chosen nanoelectrode and during the dopamine recording of the chosen nanoelectrode itself.

Based on the FM‐143 experiment, which confirmed the active release of neurotransmitters at the dopamine recording site, we were able to construct a signature dopamine current response profile from a single nanoelectrode. This signature dopamine release current approximates the neurotransmitter released from several synapses within a single SH‐SY5Y cell that engulfs the nanoelectrode. The subcellular dopamine release current has never been captured for this cell type or for single neurons. Because the dopamine response measured using CV is relative, this phenomenon suggests that the available extracellular dopamine for detection changes at the sub‐second scale when the cell is stimulated using KCl. This set of experiments demonstrated that synaptic vesicles are highly active directly adjacent to the target cell, which is releasing dopamine and producing electrical signals, suggesting that the origin of our electrochemical measurements is dopamine release as well. In the future, the signature dopamine release waveform in response to various pharmacological or electrical interrogation can be characterized by drug screening assays or biological studies.

## 3 Conclusion

We introduce Graph‐NEAs, a new class of multimodal NEA that bridge electrical and chemical signaling within electrogenic cellular networks. By integrating graphite and Nafion on platinum nanopillar electrodes, we achieve stable and sensitive electrochemical detection of dopamine from single neuron‐like cells, while simultaneously recording intracellular‐like electrical activity from neighboring sites. This integration enables co‐registered mapping of electrical excitability and neurotransmitter dynamics across multiple timescales and perturbations.

Our results reveal that dopamine release tracks sub‐ and supra‐threshold electrical activity in response to electrical stimulation, depolarization, vesicular blockade, and chronic oxidative stress. Complementary optical assays confirm that the measured chemical currents originate from active synaptic vesicle release adjacent to the sensing electrodes. We recognize several areas of future work, including micropatterning techniques [88, 89] to improve coupling ratio (# of nanoelectrodes successfully coupled to cells) and efficiency (SNR of successfully coupled nanoelectrodes), pursuing truly simultaneous, multimodal electrical, electrochemical, and optical measurements, as well as recording from both 2D and 3D cytoarchitectures of neuronal networks with larger dopamine release concentrations, such as primary neurons or dopaminergic organoids. Indeed, while the SH‐SY5Y cell line is an excellent starting point for designing proof‐of‐concept dopamine sensors, primary or iPSC‐derived dopaminergic cell lines with a well‐established record of functional dopamine exocytosis would truly allow us to test the limitations of the Graph‐NEA dopamine sensors. Furthermore, improving the selectivity of the sensor by better tuning the thickness of the Nafion coating to individual catecholamines is a key milestone in improving the Graph‐NEA platform [90].

By unifying intracellular‐like electrophysiology and real‐time electrochemical sensing within a scalable nanoelectrode platform, Graph‐NEAs provide a foundation for interrogating how electrical and chemical modalities interact to shape network behavior. Beyond fundamental neuroscience, this multimodal framework can be extended to 3D tissue models, compound screening, and closed‐loop neuromodulation systems. Ultimately, these results mark a step toward nanoscale interfaces capable of decoding and modulating the brain's hybrid electrical‐chemical language.

## 4 Methods

### 4.1 Nanopillar Electrode Array Fabrication

NEAs were fabricated as previously described in the Nano3 Cleanroom at UC San Diego [24]. Briefly, 4‐inch fused silica wafers were sonicated in acetone, followed by 2‐propanol, and dried using nitrogen gas. The fabrication schematic is shown in supplementary Figure3. The devices were spin coated with HMDS liquid KL primer and AZ1512 photoresist and exposed using the Heidelberg MLA 150 maskless aligner to create holes which were then filled with chromium using the Temescal electron beam evaporator. The wafers were then dry etched using the Oxford PlasmaLab 80 reactive ion etcher and then wet etched using 20:1 buffered oxide etch to create and shrink the nanopillars. Then, photolithography was performed again to pattern the conductive electrode pads and leads, which consisted of a 10 nm layer of Ti and 40 nm layer of Pt sputtered using the Denton Discovery 635 tool. Following lift off, a stencil was used to protect the outer pads while the wafer was passivated using 6 alternating layers of SiNx/SiOx using the Oxford Plasmalab PECVD tool, and finally, the nanopillar electrodes were cleared by first spin coating a single, thin layer of AZ1512 and wet etching the tips of the passivated pillars using 20:1 buffered oxide etch. Scanning electron microscopy (SEM) was performed after key steps using the (FEI) field electron and ion company Quanta tool to confirm nanopillar retention, uniformity, and conductive coatings.

### 4.2 Electrochemical Impedance Spectroscopy

We performed EIS for all electrodes for all devices before and after graphite/nafion coating using a bespoke setup as previously described [24]. Briefly, each nanoelectrode's outer pad was connected via a titanium wire using electrical table to a PalmSens4 potentiostat. The NEA well was filled with PBS, and an aqueous Ag/AgCl electrode was added as the reference electrode while a Pt/Ti electrode was used as the counter electrode. We performed EIS using an equilibration time of 3 s with a fixed scan, an alternating current potential of 0.25 V, and a scan frequency with 52 steps ranging from 0.1 to 5 kHz. The Nyquist plot was used to evaluate electrical contact and the impedance profile of each nanoelectrode. The Bode plot impedance value at 1 kHz was used to evaluate the nanoelectrode impedance during cell culture. A t‐test with *p*‐value <0.05 was used to detect statistically significant differences in impedance at 1 kHz before and after Nafion coating.

### 4.3 Graphite/Nafion modification of NEAs

The first layer of graphite (graphite powder, Thermos Scientific) was prepared by dispersing graphite powder in isopropyl alcohol (IPA) at a concentration of 20 mg/mL, followed by 5 min of ultrasonic mixing. This graphite layer was then deposited using electrodeposition in a two‐electrode system (platinum (Pt) was used as both the reference and counter electrode) via chronoamperometry at a constant potential of +0.5 V for 10 min. After deposition, the electrode was carefully rinsed with deionized (DI) water. The second layer of Nafion (Ion Power, inc., Nafion D520 CS Alcohol based 1000 EW at 5% weight) was also electrodeposited in the same two‐electrode setup via chronoamperometry at +0.5 V for 2 min. Following deposition, the electrode was rinsed with DI water and then baked in an oven at 80°C for 30 min.

### 4.4 Chronoamperometry of Graph‐NEAs

Chronoamperometric measurements were performed for each concentration at +0.5 V for 60 s using a two‐electrode system. The working electrode consisted of Nafion–graphite‐modified platinum (Pt) nanopillars, while a platinum wire served as both the reference and counter electrode.

### 4.5 Cyclic voltammetry of Graph‐NEAs

CV was performed to study the electrochemical behavior of the cells. The potential was scanned from 0 to 1.2 V at a scan rate of 50 V/s using a two‐electrode system. In the two‐electrode setup, a Pt reference wire served as both a counter and pseudo‐reference, which shifts the dopamine peaks to higher potentials during CV [91]. However, this setup was chosen for the sake of simplicity when a single, 1 mL well had to harbor several wire inputs for both CV and electrophysiology. The working electrode was Nafion–graphite‐modified Pt nanopillars, with a platinum wire acting as both the reference and counter electrode.

### 4.6 Graph ‐NEA Sterilization

The devices were sprayed with 70% ethanol inside their petri dishes before placing them into the BSC. The devices were wiped down using 70% ethanol on a Kim wipe, making sure not to touch the center. They were then transferred to new, sterile petri dishes. The devices were rinsed three times with autoclaved (sterile) DI water, followed by one rinse with 70% ethanol, and then rinsed once more with autoclaved DI water. They were left to dry air completely for 30. After drying, the devices were placed under UV light for 30 min.

### 4.7 SH‐SY5Y culture on Graph‐NEAs

The devices were coated with 0.02% gelatin and 5 µg/mL fibronectin extracellular matrix (ECM) mixture and incubated for 1 h at 37°C. Following incubation, the ECM was aspirated, and the devices were thoroughly rinsed with autoclaved DI water. Once the devices were ready with ECM coating, cryopreserved SH‐SY5Y cells were thawed in a 37°C water bath for 2 min, ensuring the vial's O‐ring remained above the water surface. Following thawing, the vial was sprayed with 70% ethanol and transferred into a biosafety cabinet (BSC) where all subsequent steps were performed under sterile conditions. Cells were gently added to 9 mL of pre‐warmed culture media Dulbecco's modified eagle medium/Nutrient Mixture F‐12 (DMEM: F12) with 10% fetal bovine serum (FBS) and 1% penicillin‐streptomycin (pen‐strep) in a 15 mL centrifuge tube. An additional 1 mL of media mixture was used to rinse the cryovial and recover any remaining cells. The suspension was centrifuged at 300 rpm for 6 min. After centrifugation, the supernatant was removed, and the cell pellet was resuspended in 1 mL of fresh media. A 10 µL aliquot was taken for viability and concentration assessment using a trypan blue exclusion and a Bio‐Rad TC20 automated cell counter. Based on the count, the cell suspension was adjusted to a final concentration of 2 × 10^6^ cells/mL. For seeding, 50,000 cells in 25 µL were applied to each ECM coated device and incubated for 1 h at 37°C to allow for attachment.

We used retinoic acid (RA) based differentiation of SH‐SY5Y cells. This method is widely used and yields a heterogeneous population of neuron‐like cells [77, 92, 93, 94].For this, we added 600 µL of differentiation medium having DMEM: F12 supplemented with 2% FBS, 1% pen‐strep, and 10 µM retinoic acid to each device, and cultures were maintained under standard conditions. Media was replaced after 2 days. The experiments were done on day 7.

### 4.8 Chronic BSO/Iron SH‐SY5Y culture on Graph‐NEAs

SH‐SY5Y cells were seeded on Graph‐NEAs as described above. To induce chronic oxidative stress, parallel cultures received either 100 µM ferrous sulfate (FeSO_4_) or BSO, dosed every other day for 6 days (days 1, 3, and 5). Cells were assayed on day 7.

### 4.9 HL1 culture on Graph‐NEAs

Devices were seeded following the protocol provided by Sigma–Aldrich. Briefly, devices were coated with 0.02% gelatin and 5 µg/mL fibronectin and incubated at 37°C. After coating, 20 µL of cell suspension containing 15,000 cells was seeded onto each device and incubated for 1 h to promote cell attachment. Following incubation, 600 µL of culture medium containing 87% Claycomb Basal Medium, 10% FBS, and 1% each of norepinephrine (10 mM, 100X), L‐glutamine (200 mM), and penicillin streptomycin (100X) was added to each device. The medium was changed daily, and experiments were conducted on day 7.

### 4.10 Calcium Imaging using Fluo‐4 AM dye

We used the Fluo‐4 AM Calcium Imaging Kit (Catalog no. F10489) to evaluate electrical activity in both differentiated SH‐SY5Y cells and HL1 cells during electrical recording and dopamine sensing. We followed instructions provided on the manufacturer's website. Briefly, the Fluo‐4 AM dye and its PowerLoad concentrate were combined and then vortexed with Live Cell Imaging Solution (LCIS—Catalog no. A1429DJ) that had been previously mixed with a 20 mM glucose solution. The solution was then mixed with Probenecid to prevent extrusion of dye during live imaging. This produced a working concentration of Fluo‐4 AM of approximately 1 uM. Next, we washed the cells in the NEAs with 1 mL of LCIS once, added the Fluo‐4 loading solution, and incubated for 20 min at 37°C followed by 20 min at room temperature. Finally, we removed the loading solution, washed the cells once in LCIS, and replaced with a fresh solution of LCIS along with 100 mL of the Neuro Backdrop Background Suppressor solution. We performed calcium imaging using an Olympus CKX53 Culture epifluorescent microscope accompanied by an ORCA‐Fusion Gen‐III sCMOS camera by acquiring 500 – 1000 frames with 200 – 500 ms intervals in between each frame. To analyze the videos, we manually selected regions of interest (ROIs) in ImageJ of intact SH‐SY5Y or HL1 somae and computed the average ROI intensity for every frame to obtain calcium spiking over time. Next, we computed the background fluorescence and obtained the F/F0 time traces for each cell surrounding the nanoelectrodes. We cross‐examined time‐locked electrical recordings and CV measurements to corroborate electrical active cells using multimodal data.

### 4.11 Synaptic Vesicle Imaging using FM‐143 dye

The FM‐143 dye (Catalog no. T3163) was used to quantify synaptic vesicle release on cells overlying the nanoelectrode used for dopamine sensing. In this assay, a two‐step process is used to 1) porate the plasma membrane so that FM‐143 dye will be taken up via endocytosis into the cell (brightest fluorescence intensity) and then 2) stimulate the cell to release synaptic vesicles and therefore the FM‐143 dye into the extracellular space (lowest fluorescence intensity). To perform the assay, we made a working solution of 10 µg/mL FM‐143 dye dissolved in hank's balanced salt solution (HBSS) buffer. We washed the cells once with warmed HBSS and then added 1 mL of FM‐143 working solution. Next, we stimulated the chosen nanoelectrode with an electrical stimulus delivered through the nanoelectrode itself. The stimulus consisting of biphasic square pulses going from – 500 to 500 mV, with 200 µs duration [24, 31]. We stimulated with 5 pulses, each spaced 500 ms apart. This electrical stimulus served as the first step to porate the plasma membrane of overlying SH‐SY5Y cells. After waiting for 5 min for the dye to be taken up into the cells, we washed the FM‐143 working solution out and replaced it with LCIS. We next recorded the new electrical, dopamine, and fluorescence baseline. The fluorescence images were taken with a 40x objective lens from the bottom. Finally, we added 2.5 mM KCl directly to the NEA, recorded the baseline electrical, dopamine, and fluorescence signals and then repeated this every 2 min for the following 21 min. To analyze the images, we selected ROIs of fluorescent punctae surrounding the chosen nanoelectrode using ImageJ and obtained their average F/F0 intensities over the 20 min experiment. We plotted the average intensities for all punctae for all NEAs over time in response to the KCl stimulation.

### 4.12 Scanning Electron Microscopy of SH‐SY5Y cells on Nafion‐NEAs

After fixing cells in 4% paraformaldehyde for 10 min total, samples were washed 3x with PBS and then dehydrated using a serial dilution of ethanol, at 10, 30, 50, 70, 90, and 100% consecutively. The sample was left to incubate for 5 min after each wash and then 10 min after the 70% addition. Next, the samples were left to dry at room temperature for several hours and imaged using the FEI Quanta SEM using 2 keV at high magnification.

### 4.13 Multimodal Recording Setup

Our bespoke multimodal data acquisition setup consisted of electrical recording, optical imaging of either calcium waves or FM‐143 synaptic vesicle release, and dopamine sensing using CV. Each of these modalities is described in detail below:

### 4.14 Electrical

For both SH‐SY5Y cells and HL1 cells, electrical recordings were obtained using the MultiChannel Systems (MCS) MEA2100 Mini System and MCS Experimenter, MCS Config, and MCS DataManager softwares. Except for the reserpine inhibition experiment, all recordings were conducted on a benchtop in nonsterile conditions. The NEA and (MEA) microelectrode array devices were clicked into the MCS headstage and fit with an MCS Ag/AgCl pellet reference electrode. The headstage was centered atop the Olympus CKX53 epifluorescent microscope and manually positioned to focus on the chosen nanoelectrode for dopamine sensing. The outer pad of the chosen nanoelectrode was fitted with a titanium wire and insulated with electrical tape to prevent contact from the overlying MCS headstage pin. Aluminum foil was used to block noise from the image acquisition system during recording. We used a sampling rate of 10 kHz with software filters in the MCS Config software with 0.1 Hz high pass cutoff and 3500 Hz lowpass cutoff. Files were exported to HDF5 format using the MCS DataManager software and then uploaded to Google Drive for analysis in Google Colaboratory using Python.

### 4.15 Optical

We used an inverted Olympus CKX53 epifluorescent microscope fitted with an ORCA‐Fusion Gen‐III sCMOS camera, accompanied by three cube filters for imaging transmitted light, brightfield, FM‐143 synaptic vesicle release, and Fluo‐4 AM calcium waves. The MC headstage was loaded onto an Al foil‐wrapped microscope stage, and a 40x objective lens was used to image the semi‐transparent Graph‐NEAs from the bottom. We used the Micro‐Manager image acquisition software to capture both fluorescent and brightfield images for the synaptic vesicle assay and also to capture videos of calcium waves in both the SH and HL1 cell cultures.

### 4.16 Electrochemical Detection of Dopamine

Either one or two potentiostats were used to acquire dopamine signals in real time using CV. The working electrode consisted of a titanium wire connected via an alligator clip to the potentiostat and then connected via electrical tape to the outer pad of the chosen nanoelectrode for dopamine measurements. For each potentiostat being used, the reference and counter electrode inputs were connected together and then connected to a Pt wire (either pure Pt or Ti sputtered with Pt). The Pt reference electrodes for each potentiostat were sterilized with 70% ethanol, dried with a Kimwipe, and dipped directly into the NEA well during measurements. CV was performed at several time points after different stimulation methods (electrical or drugs) ranging between 0V to 1.2 V. The fast scan CV 50 V/s with potential step of 0.2 V were used. Due to high scan rate and higher potential step, potentiostat were able collect five data points in the forward scan and raw data were smoothed. From the CV results, only the forward scan (oxidation peak) was plotted in the main figures, while the detailed steps are shown in Supplementary Figure 17a,b.

### 4.17 Electrophysiological Analysis

HDF5 files of all electrical recordings were mounted in Google Colaboratory's Python‐supporting environment. Either positive (intracellular) or primarily negative (extracellular) events recorded from either SH‐SY5Y cells or HL‐1 cells were detected using thresholds set beyond 3‐5x the standard deviation of the noise. Threshold crossings were collected with a dead time in between them related to the duration of the specific event being detected. Inter‐spike intervals, event normalization, and statistical analyses (ANOVA with Tukey's postdoc) in response to electrical stimulation and drug experiments were then performed on the waveform shapes and event indices for each class of electrical events detected.

## Supporting Information

Additional supporting information can be found online in the Supporting Information Section. **Supporting Fig. S1:** Graph‐NEA dimensions. (a) Bright field image of the device with differentiated SHSY5Y cellular network, and (b) zoom in SEM of single nanoelectrode area without any cells. **Supporting Fig. S2:** Nanopillar electrode arrays (NEA) fabrication. Schematic showing step‐bystep NEA fabrication process. The NEA fabrication process is split into two stages: Layer 1: Nanostructure fabrication, and Layer 2: Pad deposition and passivation. A design of 9 pillars with 1 micron height and 3.5 micron pitch was chosen based on previously work in our group (1) to enhance coupling with overlying neuron‐like SH‐SY5Y cells. 1. Shukla, S. *et al.* Supra‐ and sub‐threshold intracellular‐like recording of 2D and 3D neuronal networks using nanopillar electrode arrays. *Microsyst. Nanoeng.* **10**, 1–11 (2024). **Supporting Fig. S3:** Impedance and impulse response of Graph‐NEAs. a) Bode plot showing impedance spectrum before and after coating. b) Representative impulse response of single, empty nanoelectrode in media before and after coating. **Supporting Fig. S4:** Experimental timeline for device preparation, testing, and multimodal recordings. Devices were fabricated two weeks prior to experiments and coated with graphite and Nafion, followed by in vitro electrochemical validation two days before cell culture. On Day 0, cells were seeded onto the Graph‐NEAs and maintained in culture. On Day 6, multimodal recordings were performed, including electrochemical dopamine sensing, calcium imaging, and extracellular electrical activity measurements. Devices were refreshed or renewed on Day 7 to allow for subsequent experiments. **Supporting Fig. S5:** Stability. Representative chronoamperometry measurements showing stability of current response during detection of 10 µM dopamine detection using the same nanoelectrode. **Supporting Fig. S6**: Dopamine detection in media. (a) Dopamine CV in cells media. (b) Interference study Serotonin (SET), Epinephrine (EP), Nor‐epinephrine (NEP) and Dopamine (DA). **Supporting Fig. S7:** Cell viability assay: (a) Fluorescent image showing calcein‐ethidium cell viability on unmodified Pt‐NEA live cells in green dead and dead cells in red. (b) Live vs dead cells on Graph‐NEAs used for dopamine sensing. **Supporting Fig. S8:** Representative electrical recording traces before (black) and after (grey) electrical stimulation. **Supporting Fig. S9:** Supra‐and sub‐threshold effects of electrical pulse: Inter‐spike intervals before and after electrical stimulation on 3 NEAs over 3 experiments each with differentiated SH‐SY5Y cells. (a) Violin plot showing interspike intervals of action potentials before and after electrical pulse. * denotes p < 0.05 using a student's t‐test. (b) Violin plot showing interspike intervals of action potentials before and after electrical pulse. * denotes p < 0.05 using a student's t‐test. **Supporting Fig. S10:** Electrophysiological characterization of HL‐1 cells. (a) Maximum intensity projection fluorescent image after Fluo 4 addition of HL1 cells near electrode (scale bar = 30 µm). (b) CV showing Graph‐NEA response to HL1 cells firing action potentials, with a current response 5‐10x smaller than typical for SH‐SY5Y cells. (c) Calcium waveform of HL1 cells df/f before stimulation (d) Calcium wave form of HL1 cells df/f after electrical stimulation (e) Extracellular recording on Graph‐NEA. (f) Intracellular recording on Graph‐NEA. **Supporting Fig. S11:** Calcium waves in HL‐1 control and SH‐SY5Y experimental cell lines. (a) Calcium imaging video of HL1 cells on nanoelectrode. (b) Calcium imaging video of SH‐SY5Y cells on nanoelectrode. Scale bars = 35 µm. **Supporting Fig. S12:** Distribution of calcium inter‐spike intervals (ISIs) during baseline and electrical stimulation. Violin plots show the full distribution of ISIs (in seconds) recorded from SHSY5Y calcium imaging under baseline and stimulation conditions, with mean ± SD overlaid. Stimulation markedly shifted the ISI distribution toward shorter intervals, indicating increased firing activity. An independent‐samples t‐test revealed a highly significant difference between baseline and stimulation ISIs (p << 0.01), demonstrating a robust stimulation‐induced change in neuronal firing dynamics. **Supporting Fig. S13:** Supra‐ and sub‐threshold effects of KCl Stimulation. Inter‐spike intervals before and after pharmacological interrogation on 3 NEAs over 3 experiments for each drug with differentiated SH‐SY5Y cells. (a) Violin plot showing distribution of inter‐spike intervals for action potentials at different KCl concentrations. (b) Violin plots showing distribution of inter‐spike intervals for postsynaptic potentials at different KCl concentrations. (c) Violin plot showing distribution of inter‐spike intervals for action potentials at different times following reserpine addition. (d) Violin plot showing distribution of inter‐spike intervals for postsynaptic potentials at different times following reserpine addition. Astrix (*) indicates p < 0.05 following an ANOVA test with Tukey's posthoc analysis. **Supporting Fig. S14:** Dopamine response from KCl stimulation. Box and whisker plot showing dopamine response profile over time following the stimulation with potassium chloride **Supporting Fig. S15:** Upper limit of KCl stimulation. Dopamine current response of cells to various concentrations of KCl. Supporting Fig. S16: FM‐143 Controls and Statistics. Violinplot with data points (number of fluorescent punctae detected per cluster) for all conditions measured during the full FM‐143 assay, including: 1) No dye added, 2) Right after dye addition, 3) following electrical stimulation to induce dye uptake, 4) after dye washout, 5) following KCl stimulation to induce vesicle release, 6) 4 min after KCl stimulation, and 7) 12‐13 min after KCl stimulation. Asterisks indicate statistically significant differences with p< 0.05 using ANOVA with Tukey's posthoc analysis. **Supporting Fig. S17:** Electrochemical detection of dopamine by performing CV with scan rate 50 V/s and potential step of 0.2 V. In (a) the raw and smooth data is plotted. The raw data has different shape then the conventional CV due to higher potential step. The plot in (b) shows the smoothed forward scan (oxidation peak only) were plotted from (a) with potential range 0.2 to 1.2 V.

## Author Contributions

**Shivani Shukla** **, An‐Yi Chang, Anum Tahir, Joseph Wang**, and **Zeinab Jahed** conceived the project. Shivani Shukla led and performed nanoelectrode array (NEA) design, fabrication, characterization, multimodal experimental design, calcium and synaptic vesicle imaging, elec trophysiology, pharmacology studies, electrochemical sensing, data analysis, figure generation, and writing. **An‐Yi Chang** performed NEA modification with graphite and Nafion, selectivity and stability testing, electrochemical measurements, dopamine analysis, and figure preparation. **Anum Tahir** contributed to and performed NEA fabrication and device characterization and coating optimization, cell culture, calcium and synaptic vesicle imaging, electrophysiology, pharmacology experiments, electrochemical sensing, data analysis, figure preparation, and writing. **Muhammad Inam Khan** contributed to writing, schematics, electrochemical analysis, multimodal experiments, and figure preparation. **Ashley Pham** conducted SEM characterization. **Maria Reyboso, Yuma Dugas,** and **Ian McGregor** contributed to electrode fabrication and testing. **Nawab John Dar** provided cell culture and immunostaining protocols. **Noel Sebastien D Mallari** performed EIS measurements and assisted with figure preparation. **Dhivya Pushpa Meganathan** device fabrication for optimization. **Adam T. Woolley** contributed to revising the manuscript. **Joseph Wang** and **Zeinab Jahed** provided resources, funding, supervision, and contributed to writing and data interpretation.

## Funding

This study was supported by Air Force Office of Scientific Research (Grant AFOSR FA9550‐23‐1‐0090)

## Conflict of Interest

The authors declare no conflicts of interest.

## Supporting information

Supplementary Material

## Data Availability

All data and code will be made readily available at our lab's Github at https://github.com/Bionelab.
