# Supplementary material for: Chemically Selective Nanoelectrode Arrays for Real‐Time, Parallel Neurotransmitter and Electrical Recording
Source: Small Sci. 2026 Mar 28;6(4):e70249. doi: 10.1002/smsc.70249 (PMC13154913; doi:10.1002/smsc.70249)

Supplementary Information

Chemically selective nanoelectrode arrays for real-time, parallel neurotransmitter and electrical recording

Shivani Shukla[1,2,3]#, An-Yi Chang[1]#, Anum Tahir[1,4] #, Muhammad Inam Khan[1], Maria Reynoso[1], Ashley Pham[1], Yuma Dugas[1], Ian McGregor[1], Nawab John Dar[5], Noel Sebastien Dacanay Mallari[1], Dhivya Pushpa Meganathan[1], Adam T. Woolley[4], Joseph Wang[1]*, Zeinab Jahed[1,2]*

[1] Aiiso Yufeng Li Family Department of Chemical and Nano Engineering, University of California San Diego, La Jolla, CA, 92093, USA
[2] Shu Chien-Gene Lay Department of Bioengineering, University of California San Diego, La Jolla, CA, 92093, USA
[3] Chan Zuckerberg Biohub Chicago, Chicago IL, USA
[4] Department of Chemistry and Biochemistry, Brigham Young University, Provo, UT, 84602, USA
[5] Department of Cellular Neurobiology, The Salk Institute for Biological Studies, La Jolla, CA, USA

Corresponding Authors:
Joseph Wang, Zeinab Jahed, PhD

josephwang@ucsd.edu
zjahed@ucsd.edu

*Denotes corresponding authors
#Denotes co-first authors

Supplementary Figure 1. Graph-NEA dimensions. (a) Bright field image of the device with differentiated SH-SY5Y cellular network, and (b) zoom in SEM of single nanoelectrode area without any cells.

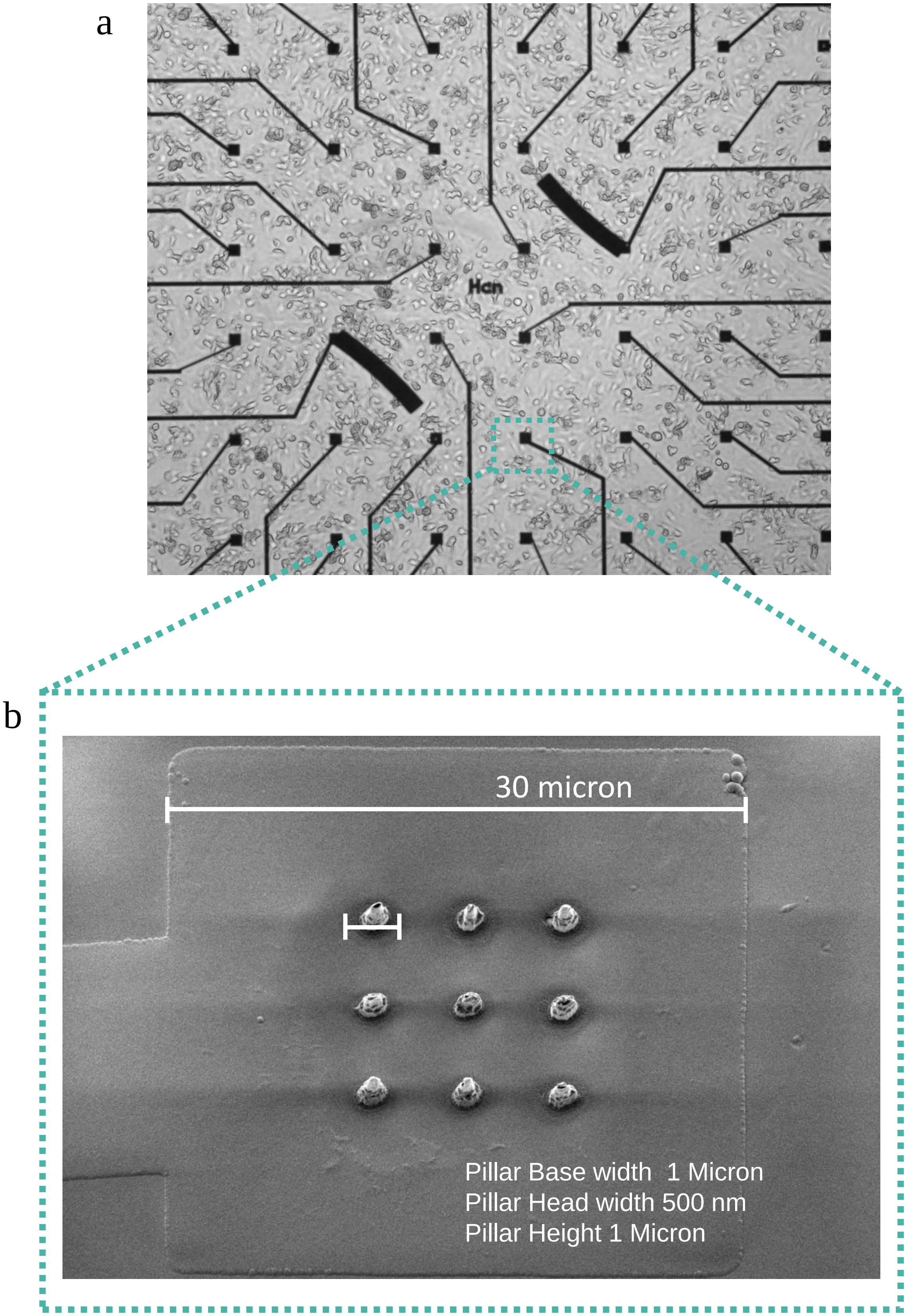

Supplementary Figure 2. Nanopillar electrode arrays (NEA) fabrication. Schematic showing step-by-step NEA fabrication process. The NEA fabrication process is split into two stages: Layer 1: Nanostructure fabrication, and Layer 2: Pad deposition and passivation. A design of 9 pillars with 1 micron height and 3.5 micron pitch was chosen based on previously work in our group (1) to enhance coupling with overlying neuron-like SH-SY5Y cells.

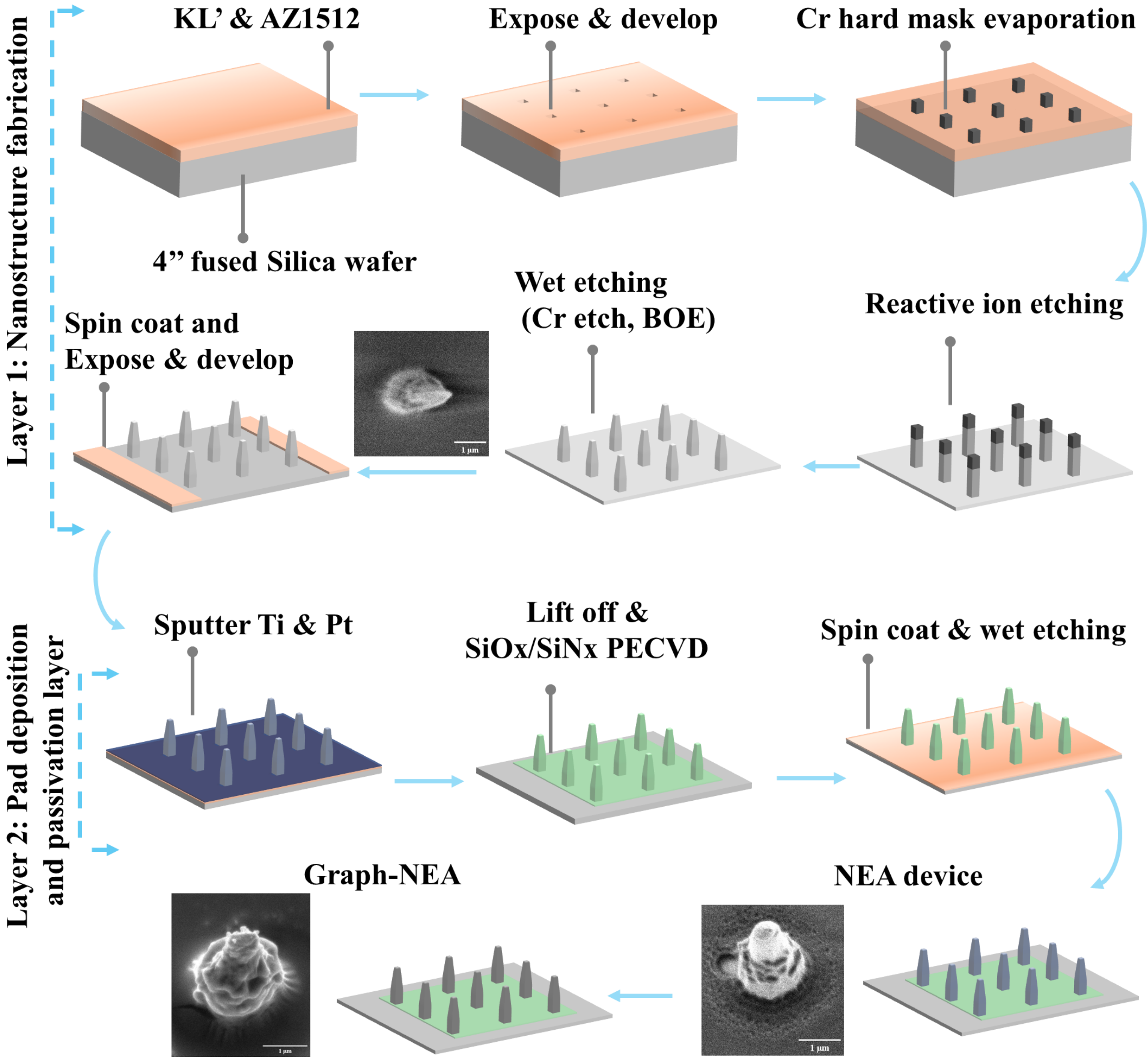

1. Shukla, S. *et al.* Supra- and sub-threshold intracellular-like recording of 2D and 3D neuronal networks using nanopillar electrode arrays. *Microsyst. Nanoeng.* **10**, 1–11 (2024).

Supplementary Figure 3. Impedance and impulse response of Graph-NEAs. a) Bode plot showing impedance spectrum before and after coating. b) Representative impulse response of single, empty nanoelectrode in media before and after coating.

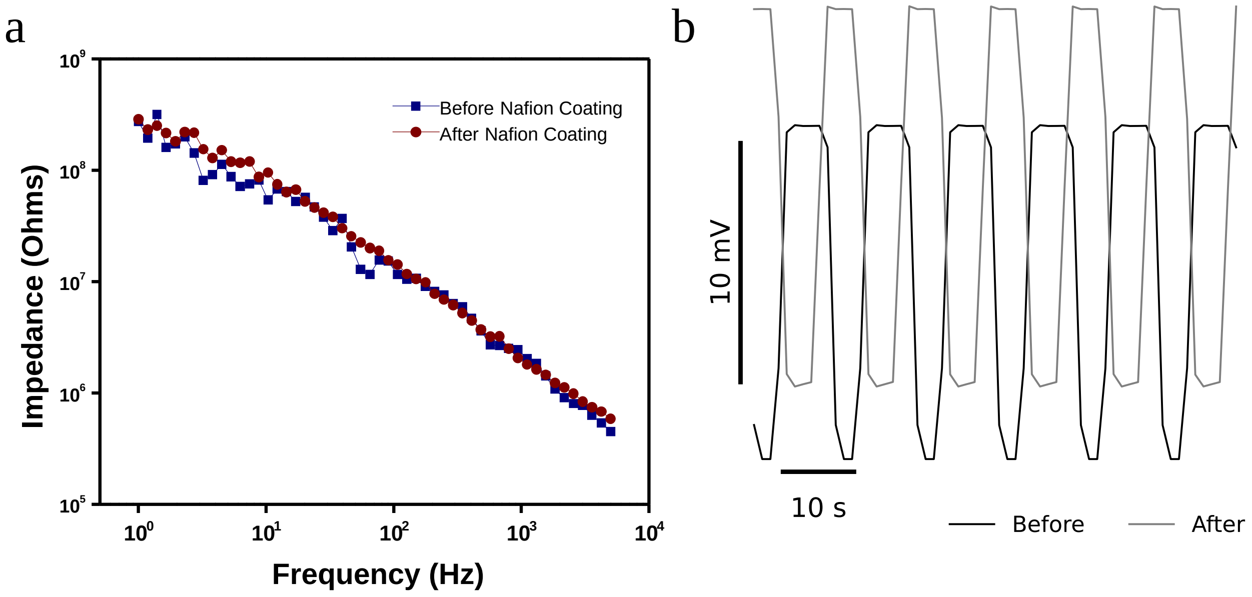

Supplementary Figure 4. Experimental timeline for device preparation, testing, and multimodal recordings. Devices were fabricated two weeks prior to experiments and coated with graphite and Nafion, followed by in vitro electrochemical validation two days before cell culture. On Day 0, cells were seeded onto the Graph-NEAs and maintained in culture. On Day 6, multimodal recordings were performed, including electrochemical dopamine sensing, calcium imaging, and extracellular electrical activity measurements. Devices were refreshed or renewed on Day 7 to allow for subsequent experiments.

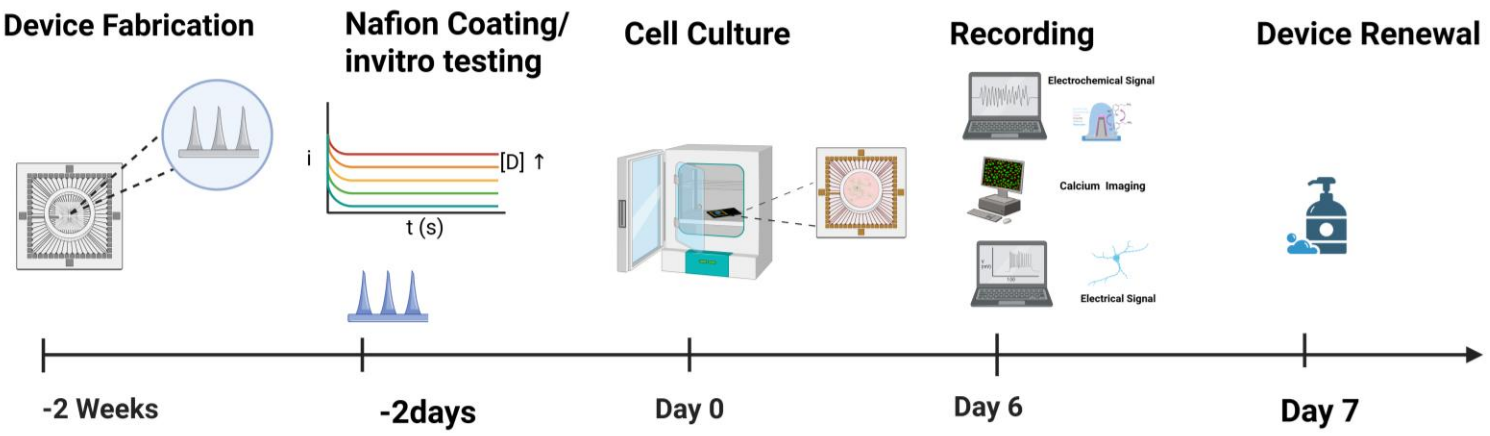

Supplementary Figure 5. Stability. Representative chronoamperometry measurements showing stability of current response during detection of 10 μM dopamine detection using the same nanoelectrode.

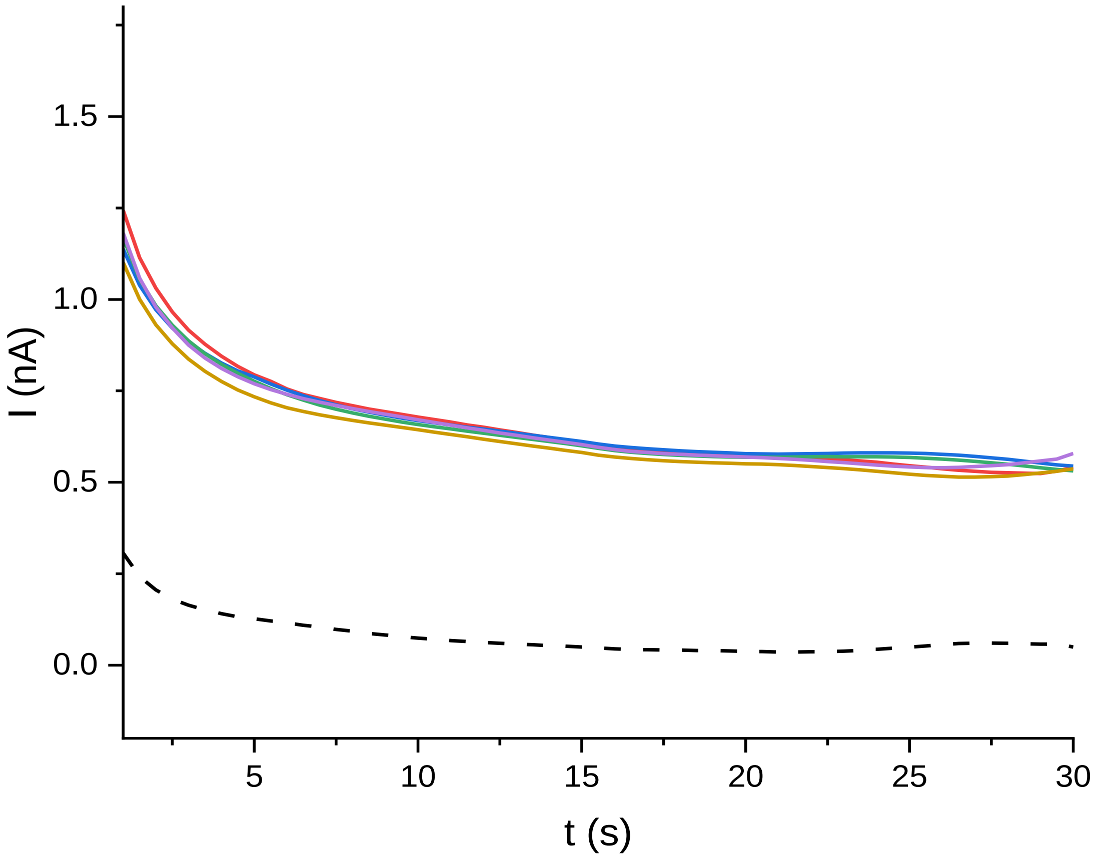

Supplementary Figure 6: **Dopamine detection in media**. (a) Dopamine CV in cells media. (b) Interference study Serotonin (SET), Epinephrine (EP), Nor-epinephrine (NEP) and Dopamine (DA)

a

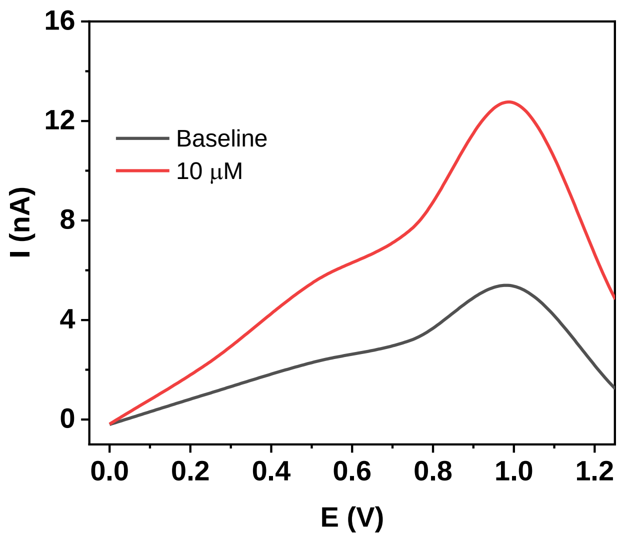

b

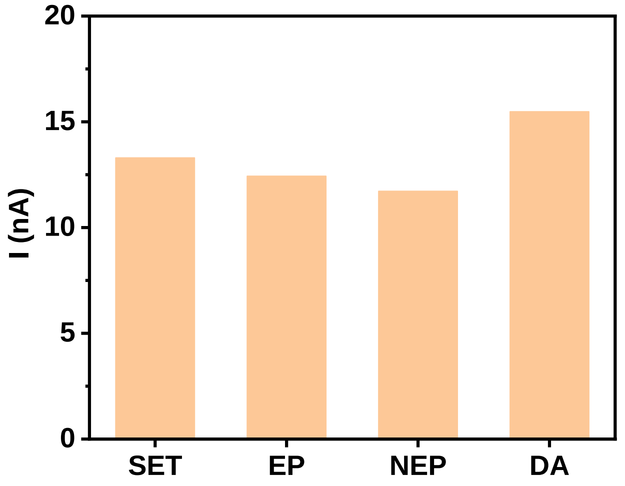

Supplementary Figure 7. Cell viability assay: (a) Fluorescent image showing calcein-ethidium cell viability on unmodified Pt-NEA live cells in green dead and dead cells in red. (b) Live vs dead cells on Graph-NEAs used for dopamine sensing.

a

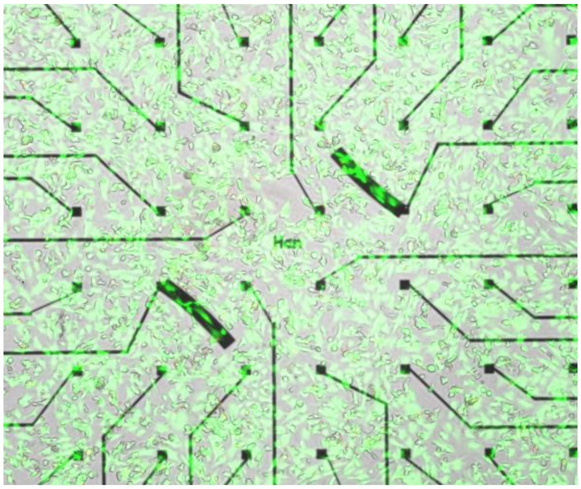

b

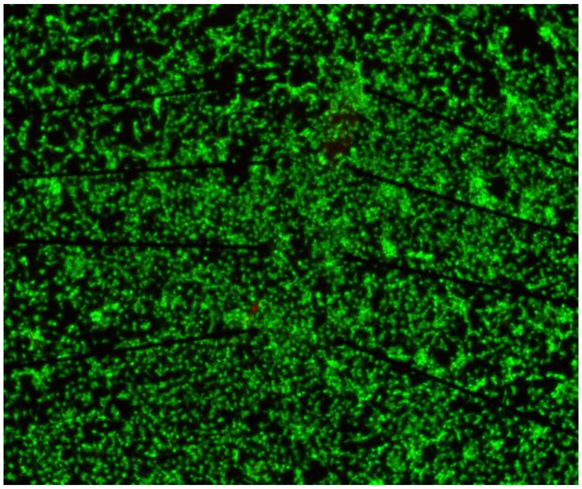

Supplementary Figure 8: Representative electrical recording traces before (black) and after (grey) electrical stimulation.

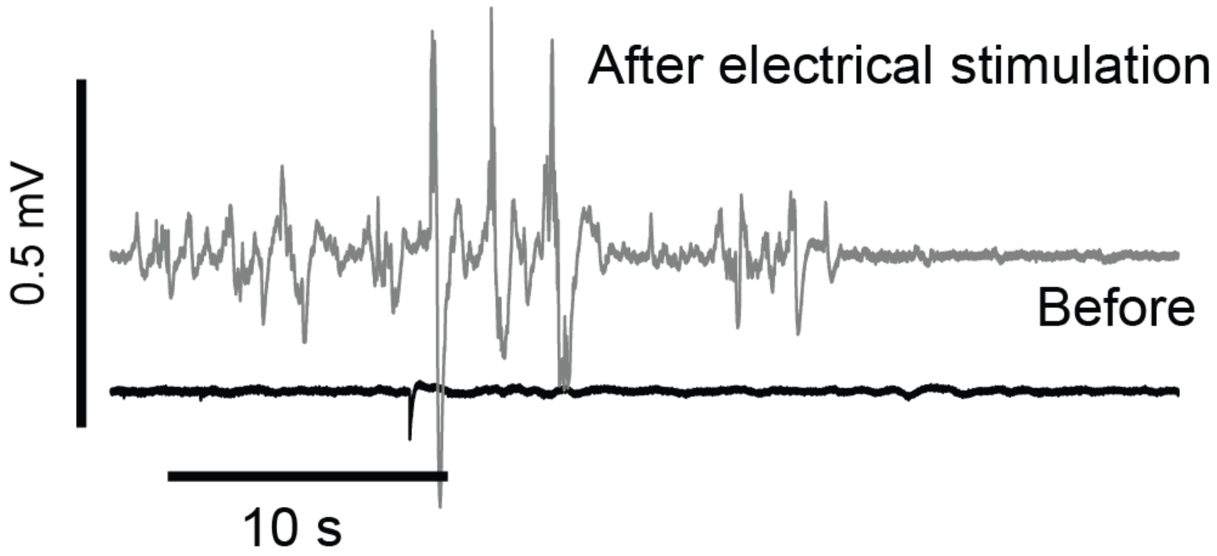

Supplementary Figure 9. Supra- and sub-threshold effects of electrical pulse: Inter-spike intervals before and after electrical stimulation on 3 NEAs over 3 experiments each with differentiated SH-SY5Y cells. (a) Violin plot showing interspike intervals of action potentials before and after electrical pulse. * denotes $p < 0.05$ using a student's t-test. (b) Violin plot showing interspike intervals of action potentials before and after electrical pulse. * denotes $p < 0.05$ using a student's t-test.

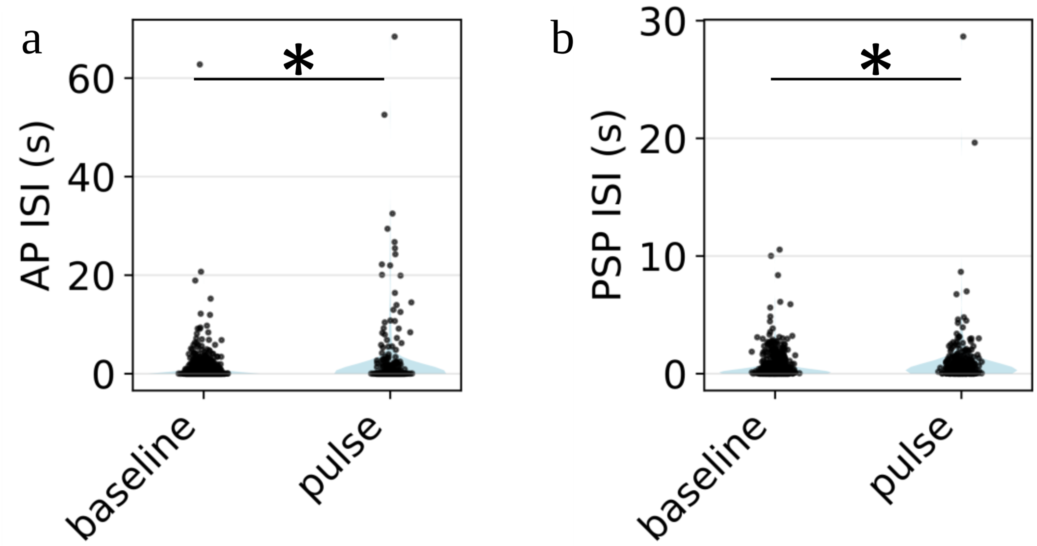

Supplementary Figure 10. Electrophysiological characterization of HL-1 cells. (a) Maximum intensity projection fluorescent image after Fluo 4 addition of HL1 cells near electrode (scale bar = 30 μm). (b) CV showing Graph-NEA response to HL1 cells firing action potentials, with a current response 5-10x smaller than typical for SH-SY5Y cells. (c) Calcium waveform of HL1 cells df/f before stimulation (d) Calcium wave form of HL1 cells df/f after electrical stimulation (e) Extracellular recording on Graph-NEA. (f) Intracellular recording on Graph-NEA.

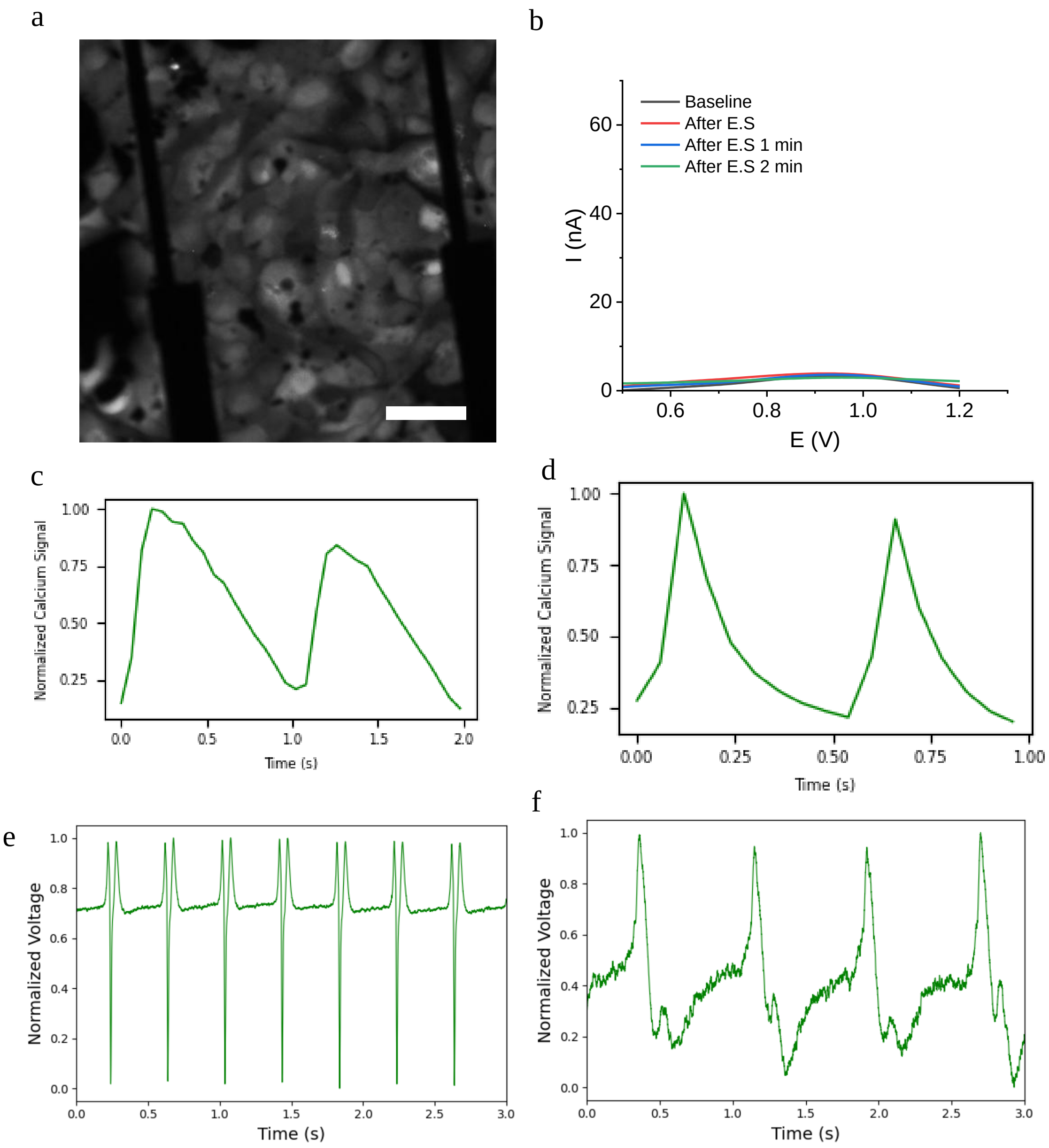

Supplementary Figure 11. Calcium waves in HL-1 control and SH-SY5Y experimental cell lines. (a) Calcium imaging video of HL1 cells on nanoelectrode. (b) Calcium imaging video of SH-SY5Y cells on nanoelectrode. Scale bars = 35 µm.

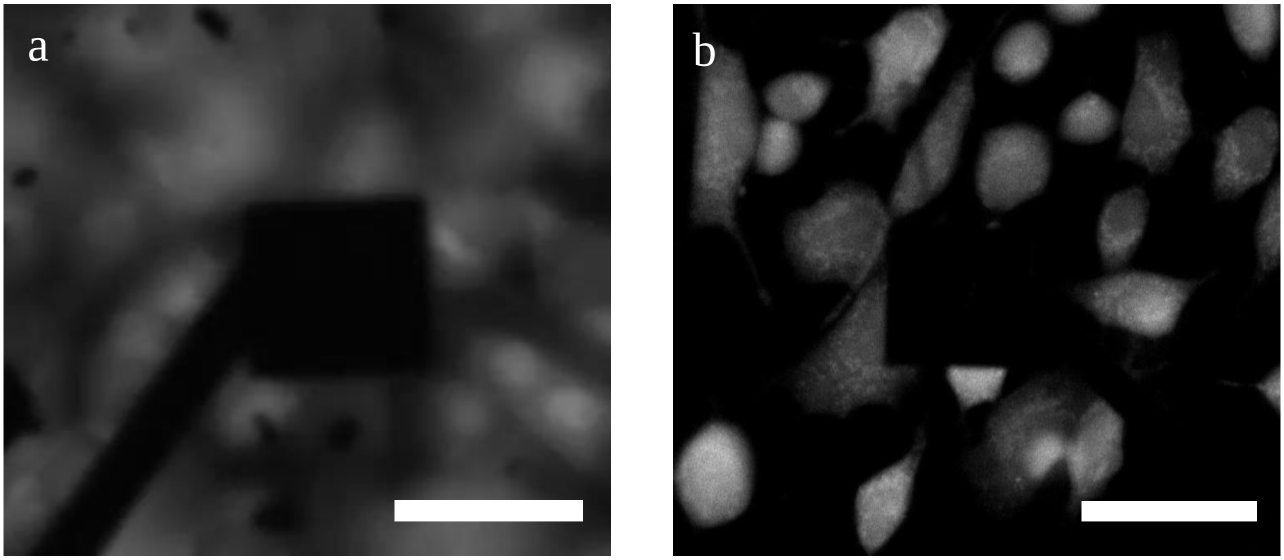

Supplementary Figure 12. Distribution of calcium inter-spike intervals (ISIs) during baseline and electrical stimulation. Violin plots show the full distribution of ISIs (in seconds) recorded from SH-SY5Y calcium imaging under baseline and stimulation conditions, with mean ± SD overlaid. Stimulation markedly shifted the ISI distribution toward shorter intervals, indicating increased firing activity. An independent-samples t-test revealed a highly significant difference between baseline and stimulation ISIs ($p << 0.01$), demonstrating a robust stimulation-induced change in neuronal firing dynamics.

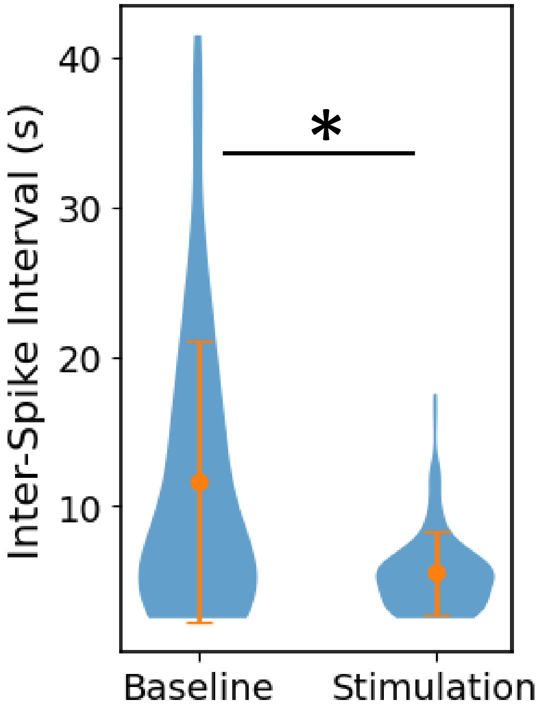

Supplementary Figure 13. Supra- and sub-threshold effects of KCl Stimulation. Inter-spike intervals before and after pharmacological interrogation on 3 NEAs over 3 experiments for each drug with differentiated SH-SY5Y cells. (a) Violin plot showing distribution of inter-spike intervals for action potentials at different KCl concentrations. (b) Violin plots showing distribution of inter-spike intervals for postsynaptic potentials at different KCl concentrations. (c) Violin plot showing distribution of inter-spike intervals for action potentials at different times following reserpine addition. (d) Violin plot showing distribution of inter-spike intervals for postsynaptic potentials at different times following reserpine addition. Astrix (*) indicates $p < 0.05$ following an ANOVA test with Tukey's posthoc analysis.

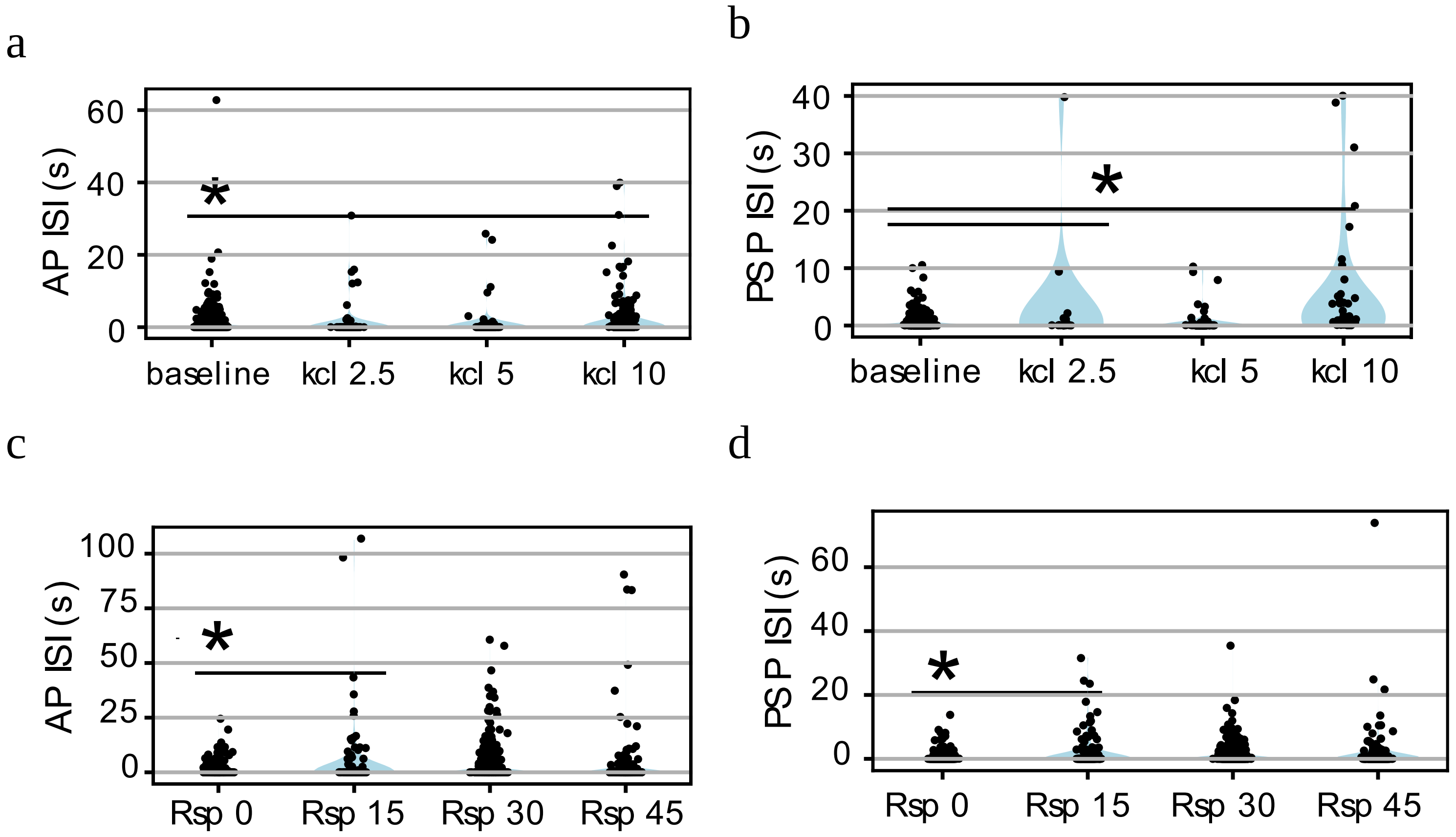

Supplementary Figure 14. Dopamine response from KCl stimulation. Box and whisker plot showing dopamine response profile over time following the stimulation with potassium chloride

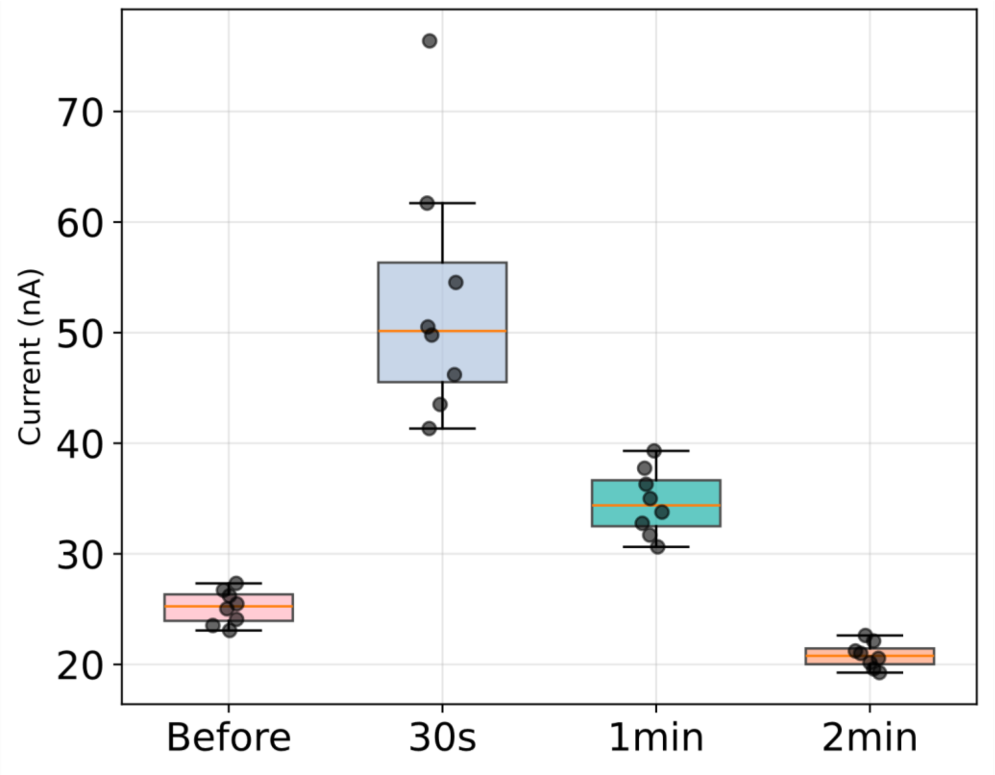

Supplementary Figure 15. Upper limit of KCl stimulation. Dopamine current response of cells to various concentrations of KCl

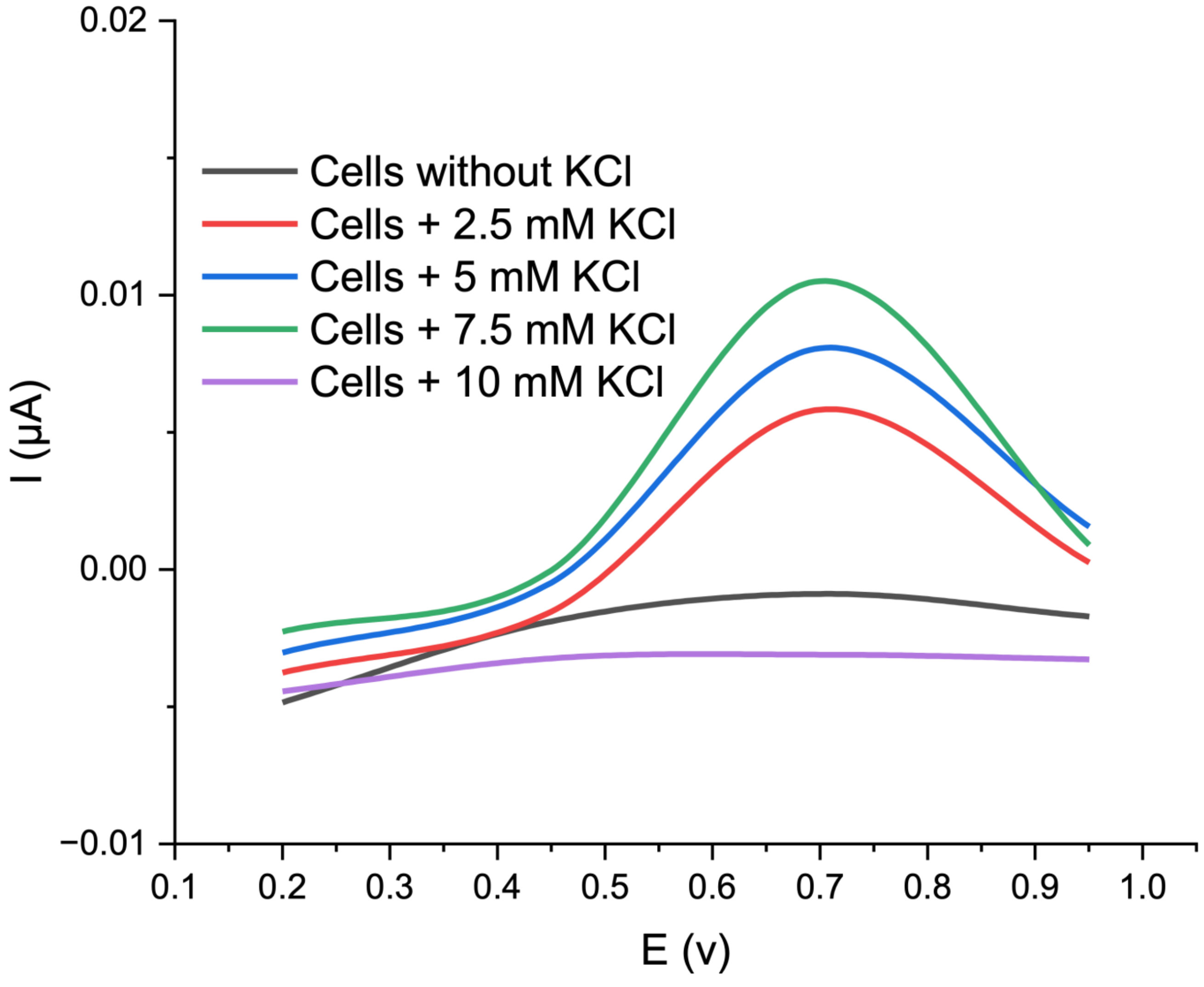

Supplementary Figure 16: **FM-143 Controls and Statistics.** Violin plot with data points (number of fluorescent punctae detected per cluster) for all conditions measured during the full FM-143 assay, including: 1) No dye added, 2) Right after dye addition, 3) following electrical stimulation to induce dye uptake, 4) after dye washout, 5) following KCl stimulation to induce vesicle release, 6) 4 min after KCl stimulation, and 7) 12-13 min after KCl stimulation. Asterisks indicate statistically significant differences with $p < 0.05$ using ANOVA with Tukey's posthoc analysis.

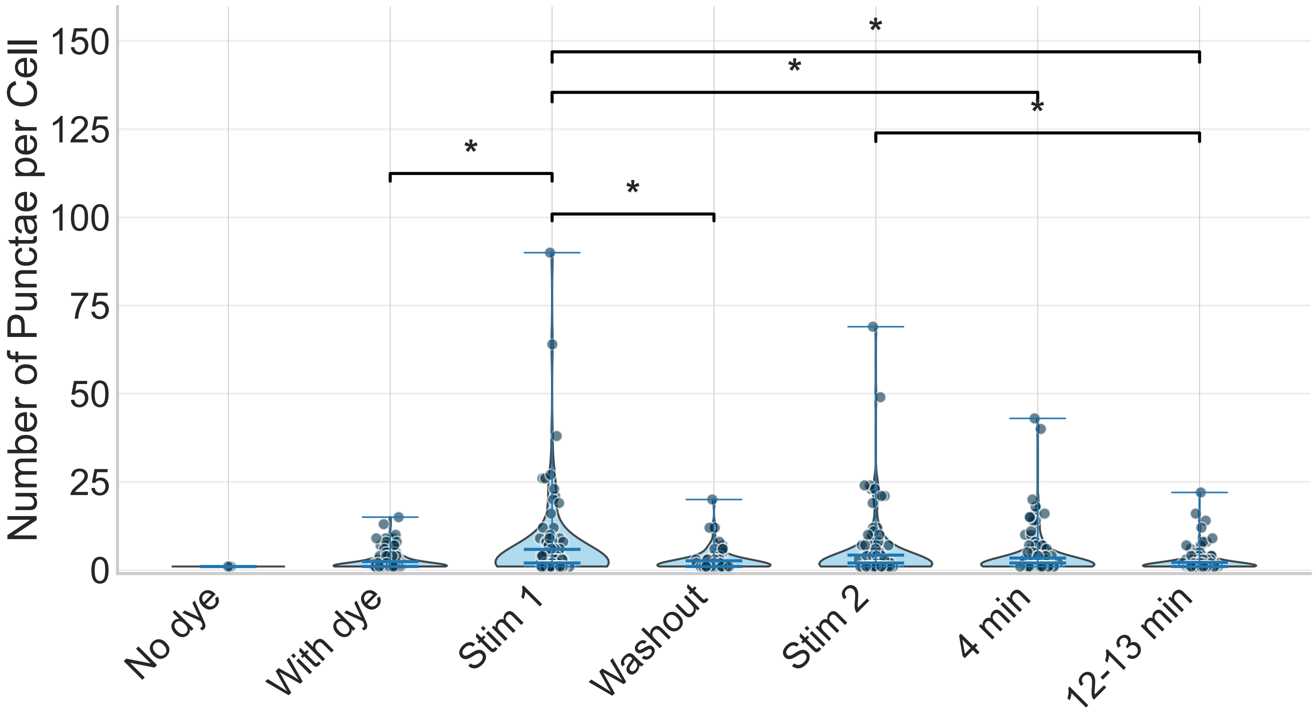

Supplementary Figure 17. Electrochemical detection of dopamine by performing CV with scan rate 50 V/s and potential step of 0.2 V. In (a) the raw and smooth data is plotted. The raw data has different shape then the conventional CV due to higher potential step. The plot in (b) shows the smoothed forward scan (oxidation peak only) were plotted from (a) with potential range 0.2 to 1.2 V.

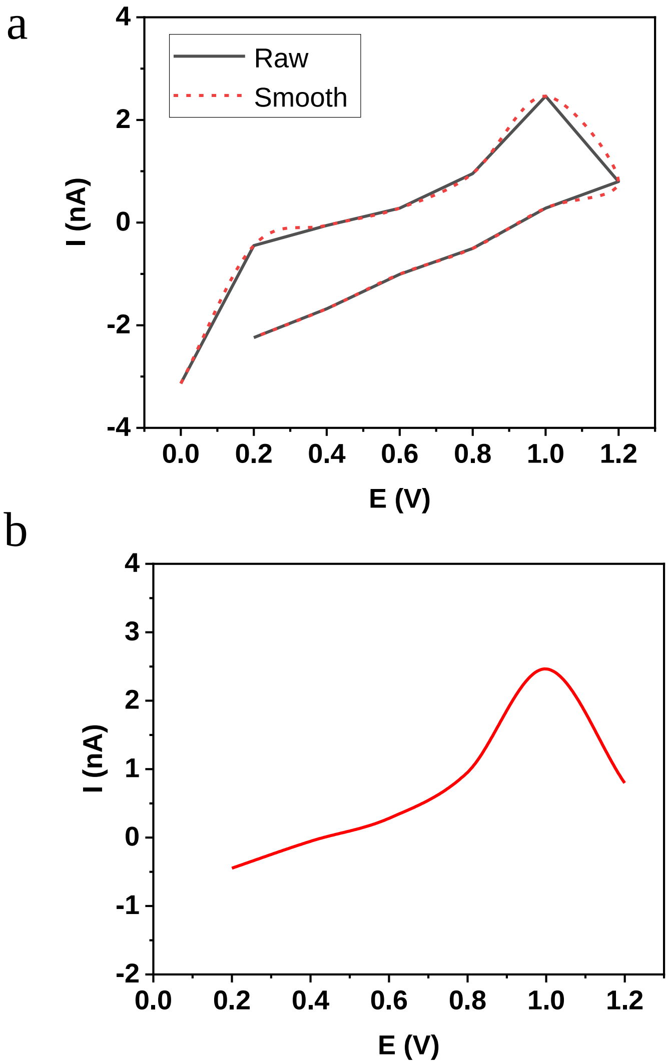

Supplement: Supplementary file 1 — Supplementary Material [file SMSC-6-e70249-s001.pdf]
